# Overcoming Stability Hurdles of Transition Metal Phosphides for H_2_ Evolution: A Review

**DOI:** 10.1002/cssc.202501388

**Published:** 2025-09-11

**Authors:** Anelisse Brunca da Silva, Eduardo Arizono dos Reis, Caue Ribeiro, Lucia Helena Mascaro

**Affiliations:** ^1^ Departament of Chemistry Universidade Federal de São Carlos Rod. Washington Luiz, Km 235 São Carlos 13565‐905 SP Brazil; ^2^ National Nanotechnology Laboratory for Agribusiness (LNNA) EMBRAPA EMBRAPA Instrumentation XV de Novembro 1452 São Carlos 13561‐206 SP Brazil; ^3^ Chemistry Institute of São Carlos University of São Paulo – USP Av. Trabalhador São Carlense 400 São Carlos 13563‐120 SP Brazil

**Keywords:** hydrogen evolution reaction, metal phosphides, stability

## Abstract

The necessity of efficient electrolyzers for H_2_ production has boosted the number of publications on the development of highly active and stable electrocatalysts. Recently, transition metal phosphides (TMPs) have been reported as excellent materials for H_2_ electrochemical production. Though most publications demonstrate the applicability of Co, Mo, Fe, and Ni‐based TMPs in a wide pH range, there's a lack of consolidated information about the material's durability versus pH, especially in harsh environments (e.g., high acidity or alkalinity and seawater). The main purpose of this review is to correlate the advancement of knowledge about the TMPs electronic and physical‐chemical properties and the improvement of hydrogen evolution reaction in a wide range of pH and salinity to present some meaningful perspectives for the near future development.

## Graphical abstract

Developing durable electrocatalysts for water splitting remains a major challenge. This review examines strategies to enhance the long‐term stability of earth‐abundant transition metal phosphides for hydrogen evolution. It covers advances in material design and modification across pH conditions and includes stability analyzes in practical electrolyzer systems, highlighting effectiveness and challenges.

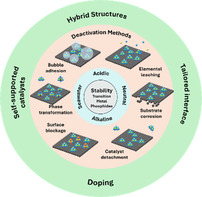

## 1 Introduction

The aggravation of the environmental and fossil energy crisis and the limited supplies of fossil fuels reinforce the urge to transform how energy is produced, transported, and consumed.^[^ ^1^ ^]^ Cleaner energy sources are essential to achieve the CO_2_‐net zero emission target by 2050.^[^ ^1^, ^2^, ^3^ ^]^ The intermittency of these renewable energy sources restricts energy delivery efficiency. Hydrogen, a high‐density energy vector, is a key molecule for transitioning to a sustainable energy system.^[^ ^4^, ^5^, ^6^ ^]^ Developing hydrogen energy based on water electrolyzers through renewable electric energy and the hydrogen fuel cell is expected to be the most promising solution for long‐term sustainability.^[^ ^6^, ^7^ ^]^

Electrochemical splitting of the water molecule is an efficient approach to producing highly pure hydrogen with zero carbon footprint, and its effectiveness depends mainly on the electrocatalyst‐electrolyte interface. Currently, platinum group metals (PGM) catalysts have the most remarkable performance in H_2_ production over a wide pH range. However, their long‐term commercialization is hindered by their scarcity, high price, and unsatisfactory performance.^[^ ^8^, ^9^, ^10^ ^]^ The high costs of the electrolysis devices compromise their industrial application; up to now, the H_2_ production by electrochemical devices represents only 4%–5%.^[^ ^11^, ^12^ ^]^ Designing nonnoble metal electrocatalysts with long‐term stability and industrial‐relevant current density has become a significant interest in making water‐splitting technology economically more appealing.^[^ ^13^ ^]^ Furthermore, large‐scale water electrolysis technologies must regard freshwater feedstock and rationally design suitable electrocatalysts to operate efficiently in pH‐universal media and enable the utilization of other water sources such as seawater, industrial wastewater, and residential water.

Platinum‐based electrocatalysts, while serving as the benchmark for hydrogen evolution reaction (HER) in acidic media^[^ ^14^ ^]^ with their durability and long‐term stability exceeding 100 h,^[^ ^15^ ^]^ are still hampered by their restricted stability in acidic environments and high costs.^[^ ^14^, ^16^ ^]^ An essential requirement for practical device performance is underscored by the necessity to develop cost‐effective electrocatalysts with improved activity and stability.^[^ ^17^ ^]^ Over the past decade, many efforts have been made to develop nonnoble transition metal‐based electrocatalysts for water splitting, especially Ni‐, Co‐, Fe, and Mo‐ based materials.^[^ ^18^, ^19^, ^20^, ^21^ ^]^ TMPs have emerged as one of the most promising classes of earth‐abundant electrocatalysts for HER. TMPs are well‐known catalyst materials for the hydrotreating process, such as hydrodeoxygenation (HDO), hydrodenitrogenation (HDN), and hydrodesulfurization (HDS).^[^ ^22^, ^23^, ^24^ ^]^ The similarity between the hydrogen sorption/desorption steps for HDS and HER indicated that metal phosphides could be active catalysts for HER.^[^ ^25^ ^]^ Liu and Rodriguez^[^ ^26^ ^]^ predicted by Density Functional Theory (DFT) calculations that Ni_2_P is a promising catalyst for the hydrogen evolution reaction. In 2013, the predictions were validated by experimental studies;^[^ ^25^ ^]^ thenceforward, other transition metal‐based phosphides, CoP, MoP, and FeP, have been explored as catalysts for the HER.^[^ ^27^, ^28^, ^29^, ^30^ ^]^

Metal phosphides possess a distinct electronic structure derived from the negatively charged phosphorous atoms that weaken the metal–hydrogen bond strength and stimulate H desorption, hence HER performance.^[^ ^31^, ^32^ ^]^ A minor variation of the metal‐phosphorous stochiometric ratio is enough to modify TMP's electronic structure and catalytic properties. Regarding their composition, these materials can be classified as Metal‐rich and Phosphorus‐rich phosphides. Metal‐rich TMPs have metallic characteristics similar to noble metals, with excellent electrical conductivity due to the high delocalization of valence electrons through M–M bonds.^[^ ^33^ ^]^ In contrast, P‐rich TMPs are likely to present semiconducting or insulating behavior but have higher chemical stability.^[^ ^34^, ^35^ ^]^ Finding the appropriate ratio of P amount is essential for designing high‐performance electrocatalysts; for example, Ni‐P with high P‐content (NiP_2_) presents enhanced HER catalytic activity over lower P‐content catalysts (Ni_2_P and Ni_5_P_4_).^[^ ^36^ ^]^ Similar behavior was observed for Mo_3_P and MoP.^[^ ^37^ ^]^ TMPs have also shown excellent anticorrosive features in a wide range of pH^[^ ^38^, ^39^, ^40^ ^]^ caused by the unique interactions between P‐Metal. For Ni phosphides, corrosion resistance is intrinsically associated with the proportion of Ni and P content.^[^ ^41^ ^]^ For this type of transition metal phosphide, a lower P percentage is sufficient to improve the stability of the catalyst in both alkaline and acidic mediums,^[^ ^42^ ^]^ performing higher corrosion resistance than other Ni‐based electrocatalysts, such as pure Ni and Ni‐Mo alloys and sulfides.

Moreover, the variety of chemical and structural compositions possesses a wide variety of possible structures with distinct physicochemical properties.^[^ ^43^, ^44^, ^45^ ^]^ Recently, researchers have been focused on developing strategies for improving TMPs performance and the adsorption strength of intermediates.^[^ ^46^ ^]^ Morphology design control, surface engineering (hydrophilicity, active sites exposure, and defect engineering), doping, alloying, amorphization, and heterointerface construction have been explored to optimize the metal phosphides’ efficiency for HER.^[^ ^47^, ^48^, ^49^, ^50^, ^51^ ^]^ TMPs have many survey reviews in the literature focused on catalytic activity,^[^ ^52^, ^53^ ^]^ nanostructuration,^[^ ^54^, ^55^, ^56^, ^57^ ^]^ design,^[^ ^58^, ^59^ ^]^ and synthetic strategies.^[^ ^45^, ^54^, ^60^, ^61^ ^]^ However, its long‐term stability for HER has not been explored in detail. Even though some excellent review articles have recently been published,^[^ ^62^, ^63^ ^]^ they address several types of electrocatalysts, and there is still a lack of substantial discussion and critical analysis on how the TMPs’ design and tailoring strategies influence intrinsic activity and long‐term stability in a specific pH environment.

**Figure 1:**
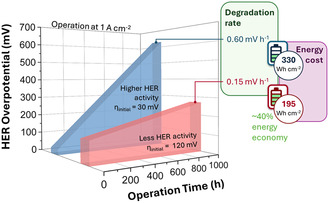
Activity–stability trade‐off in HER at 1 A cm^−^^2^: stable catalyst (red) cuts energy cost by ≈40% after 1,000 h vs. fast‐degrading one (blue).

TMPs have proved to be efficient catalysts for HER with outstanding electrical and mechanical properties, but achieving the long‐term stability required for industrial applications is still necessary.^[^ ^64^ ^]^ Long‐term performance is more valuable in practical industrial applications than activity when considering the energy cost. **Figure** **1** illustrates this concept with two representative HER catalysts. The first exhibits a lower initial overpotential of 30 mV. Still, it degrades rapidly at a rate of 0.6 mV h^−1^, and the second one, while starting with a higher overpotential of 120 mV, degrades much more slowly at 0.15 mV h^−1^. Over a 1,000 h operational period, the more stable catalyst, despite its initially higher overpotential (four times that of the first), resulted in energy savings of up to 40%.

Ideally, a high catalytic activity must be accompanied by significant durability and stability under harsh operating conditions.^[^ ^65^ ^]^ Thus, it is necessary to develop strategies to improve long‐term stability while maintaining the efficiency of TMPs in H_2_ evolution from water‐splitting.^[^ ^66^, ^67^ ^]^ Since TMPs are mature enough to have an in‐depth survey exploring that aspect, the primary purpose of this review is to provide an overview of the advancement of knowledge about the TMP's electronic and physical‐chemical properties and the improvement of the long‐term stability for HER in harsh (acidic and alkaline), neutral, and seawater conditions to present some meaningful guidelines for the near future development and transitioning from laboratory to industrial applications.

## 2 Key Descriptors for HER Performance

### 2.1 Activity Descriptors

Identifying the factors governing HER catalytic activity across different pH environments is crucial for designing efficient electrocatalysts. The volcano plot offers an insightful framework for interpreting HER activity, indicating that a suitable catalyst should exhibit a thermo‐neutral Δ*G*_H_.^[^ ^47^, ^68^, ^69^ ^]^ For transition metal phosphides (TMPs), this principle helps in predicting the TMPs intrinsic activity trend in acidic media.^[^ ^68^, ^70^ ^]^ As an example, Zhang and coauthors^[^ ^71^ ^]^ have demonstrated through computational calculations that there is a solid composition dependance in the H‐adsorption energy for Ni−P compounds. As the P content increases the Δ*G*_H_ becomes more distant from zero owing to the electrons transferring from the P atom to H, which prevents the H‐adsorption. The optimized Ni_2_P (111) structure possesses a near zero Δ*G*_H_ for hydrogen of −0.012 eV being an ideal electrocatalyst for HER. Mueller and Li^[^ ^32^ ^]^ studied by Monte Carlo simulation the influence of hydrogen coverage and interactions between the active site atoms of cobalt phosphides. The authors verified that the Cobridges in Co_2_P are the preferential sites for H‐atom adsorption. The authors performed the cluster expansion model for understanding the HER mechanism of the Co surface planes. Unlike Pt, the rich‐Co phosphide presents a minor energy barrier for the Heyrovsky step, favoring the Volmer–Heyrovsky mechanism over the Volmer‐Tafel pathway for the HER.

Furthermore, as hydrogen coverage increases, the hydrogen adsorption‐free energy is weakened owing to strong interactions between the H_ads_ in neighboring metal bridges, significantly lowering the Tafel coefficient. Other reported computational studies^[^ ^72^ ^]^ revealed that unsaturated surface atom defects in metal‐ or phosphorous‐deficient structures could tune the catalytic activity of TMPs toward HER. These studies reinforce that the energy binding theory is a helpful tool for identifying the hydrogen adsorption ability of the materials; however, it is too simplistic to deeply describe and understand the activity trends of HER in more complex systems, such as neutral, alkaline, and seawater media.

The HER is extremely pH‐dependent, a fact that is reflected in the reactant species and the whole local electrochemical environment surrounding the catalyst's active sites. In acidic electrolytes, the HER kinetics are significantly higher than in alkali environments. However, in higher pH conditions, the H_ads_ formation depends on the water dissociation step, which requires extra energy input. This extra energy barrier implies a sluggish Volmer step, which is the key to understanding the rate‐determining step for the HER. Moreover, the role of adsorbed OH in the alkaline HER mechanism remains poorly understood. The (oxy)hydroxy coverage in metallic‐based catalyst surfaces may also influence the hydrogen adsorption/desorption process since the H atoms are not primarily in contact with the metallic atom's sites.^[^ ^73^, ^74^ ^]^ Recently, significant advances have been made in investigating these key factors that determine the slow HER kinetics in alkaline media, and several theories were proposed,^[^ ^70^, ^75^ ^]^ including water dissociation theory,^[^ ^76^ ^]^ interfacial water reorganization,^[^ ^77^ ^]^ and hydroxide binding theory.^[^ ^78^ ^]^

In contrast with extreme pH conditions, the knowledge of an accurate activity descriptor for HER under (near‐)neutral conditions is far behind. The swing in the local pH near the electrode surface can be considered one of the biggest challenges for retaining HER activity in neutral media.^[^ ^79^, ^80^ ^]^ Shinagawa and Takanabe^[^ ^80^ ^]^ proposed that, for buffered neutral systems, the perturbation of the dehydration of the reactant and the electrode surface is the key to improving the HER performance. Therefore, the neutral HER depends on the electrolyte viscosity, ionic sizes, and the influence of the electric field on the double‐layer structure of the electrode interface.^[^ ^81^, ^82^ ^]^ Nevertheless, to date now, there is no quantitative explanation of how the factors mentioned above compromise the HER kinetics for more complex environments.^[^ ^70^ ^]^

### 2.2 Stability Descriptors

The stability or durability of the electrocatalyst is the most crucial parameter for determining the material's performance and enabling its application in electrolyzers. Stability in electrochemical response is typically evaluated using methods such as constant potential (CP), which is represented by the I−t curve, and constant applied current (CA), represented by the E−t curve, as well as cyclic voltammetry (CV) tests. Typically, CP is performed at the potential required for −10 or −100 mA cm^−^^2^, and CA at these current densities. CV tests are used to accelerate electrode deterioration, typically involving with at least 1,000 cycles. However, considering the shared goal of developing earth‐abundant catalysts that are competitive for electrolyzer applications, evaluating the durability of materials using low current densities such as −10 mA cm^−^^2^ and short electrolysis periods is insufficient. Furthermore, considering the use of these catalysts in systems coupled with contaminant remediation or as cathodes in hybrid photoelectrochemical systems, it is fundamental to evaluate the electrocatalyst's durability at a lower current density range. This type of stability assessment may be enough for an initial screening during catalyst development and even for comparison with other electrodes described in the literature. The electrocatalyst's suitability and durability in electrolyzers are some factors that must be considered, such as applied current density, long‐term stability tests, operating temperature, and the electrolyte concentration specific to each system.

Moreover, since power fluctuations, system shutdowns, and startup cycles can affect the catalyst's active sites, the catalyst stability and performance in noncontinuous operation should be examined, especially in systems powered by intermittent power supplies. Therefore, assessing catalyst stability in both modes is essential for a thorough evaluation of how the catalyst will perform in real‐world applications.

To simulate such dynamic operating conditions and evaluate long‐term stability, several electrochemical accelerated stress tests have been developed for water electrolyzers.^[^ ^83^, ^84^, ^85^ ^]^ Potential cycling between high and low potentials is one of the most used approaches for assessing stability. The shutdown and startup steps of potential cycling tend to promote severe degradation. Recent studies^[^ ^86^, ^87^ ^]^ have shown that the cathode side tends to contribute more significantly to variations in open‐circuit voltage during potential cycling, indicating an asymmetrical degradation behavior between the electrodes in the electrolyzer systems.

Based on the simulated solar profiles, combining constant and pulsed currents, high current densities, and extended durations contribute significantly to performance loss. Su et al.^[^ ^88^ ^]^ have investigated narrow‐range current density fluctuations applied in short step times, observing greater degradation compared to longer steps, even under wider current density variations. In addition to these cycling protocols, other techniques such as electrochemical impedance spectroscopy and polarization curve tracking are often used to monitor the evolution of charge‐transfer resistance, mass transport limitations, and kinetic losses throughout the testing period.^[^ ^85^, ^87^, ^89^, ^90^ ^]^ The mentioned electrochemical tests enable the analysis of the operational limits and durability of electrolyzer components, helping to guide the development of more robust materials and system designs for future energy applications. In addition to evaluating electrochemical stability, comprehensive physical characterization, and pre‐ and post‐stability tests are essential to elucidate catalyst degradation.^[^ ^91^, ^92^ ^]^ Changes in morphology, phase, and valence after long‐term electrolysis are typically probed using techniques such as scanning electron microscopy – SEM, energy dispersive spectroscopy – EDX mapping, transmission electron microscopy – TEM, X‐ray diffraction –XRD, Raman spectroscopy and X‐ray photoelectron spectroscopy – XPS). For metal phosphide electrocatalyst, inductively coupled polasma optical emission spectroscopy (ICP‐OES) analysis of the postelectrolysis electrolyte is essential to verify any metals and P dissolution. The combination of these postcharacterization analyzes is useful to indicate whether the electrode deteriorated (by leaching, physical material loss) or if any restructuring occurred during operation.

Despite their utility, these techniques do not offer insights into the deactivation or structural‐phase change mechanisms. To achieve this goal, in situ techniques such as XRD, Raman spectroscopy, X‐ray absorption spectroscopy (XAS), X‐ray absorption near‐edge structure (XANES), and extended X‐ray absorption fine structure (EXAFS) are invaluable tools for monitoring the interfacial electrocatalysts dynamics and possible structural changes. Utilizing in situ characterization techniques is an important strategy that enables crucial insights into the catalyst structure, composition, and morphology in operational conditions for comprehensively understanding the TMP's electrocatalyst behavior and degradation pathways in long‐term operation. This understanding is essential for advancing the design of more robust electrocatalysts capable of maintaining catalytic activity under operational conditions.

The lack of a standardized metric for stability makes it challenging to compare electrode performance in terms of durability, unlike catalytic activity, which can be addressed using the overpotential values at specific current densities (e.g., 10 and 100 mA cm^−2^). To address this, Cherevko et al.^[^ ^65^ ^]^ proposed the stability number (*S*), a parameter that relates the amount of reaction product (H_2_ or O_2_) generated to the molar quantity of dissolved ions from the catalyst. A higher *S*‐number indicates higher electrode or material stability, as it reflects less dissolution of active sites during operation. Notwithstanding the fact that the *S*‐number is primarily considered for dissolution as the main deactivation mechanism, this parameter has become a widely adopted metric for evaluating the stability of transition metal catalysts. The application of the S‐number as a comparative tool serves as a valuable complement to traditional metrics of catalytic activity. However, this parameter itself is not applicable for other deactivation mechanisms, such as structural reorganization or interfacial phase transitions, limiting its scope as a universal stability descriptor.

Although computational methods and tools have advanced, predicting material stability through theoretical calculations is less efficient and less developed compared to predicting catalytic activity. In this context, the Pourbaix diagram is an important tool to evaluate material stability in aqueous environments by tracking the chemical potential of species as a function of the electrolyte pH. However, the Pourbaix diagram is based on thermodynamic equilibrium and does not consider open circuit potential dissolution and corrosion. Recently, the development of more accurate Pourbaix diagrams made it possible to progress in theoretical and computational modeling methods, making them a more reliable tool for the prediction of the transformations of various electrocatalysts. For instance, Nørskov's group^[^ ^93^ ^]^ developed a SCAN (strongly constrained and appropriately normed) functional‐based approach to construct the Pourbaix diagrams of MoP and CoP, assessing their stability under hydrogen evolution reaction conditions. DFT calculations were employed to estimate the decomposition‐free energy of these materials, identifying stable chemical species within the Pourbaix diagram. At highly negative potentials (<−0.50 V vs. RHE), self‐passivation and high energy barriers for solid‐solid transitions prevent material decomposition, whereas, at positive potentials (≈1.0 V vs. RHE), both materials undergo complete corrosion. Moreover, significant dissolution was observed for Co‐ and Mo‐ phosphides at their Open circuit potential (OCP) (0.11 V and 0.38 V vs. RHE, respectively), which was attributed to the substantial thermodynamic driving force for decomposition. This study not only emphasizes the importance of the Pourbaix diagram for predicting electrocatalyst stability but also demonstrates the need for catalysts with low OCP values or ensuring the application of maintaining minimum potential to the electrochemical system to prevent dissolution during the shutdown steps. Although still in its early stages, this data‐driven approach shows great promise to complement traditional methods and accelerate the development of durable electrocatalysts.^[^ ^94^, ^95^, ^96^, ^97^ ^]^

## 3 Root Causes of TMP Performance Loss During HER

**Scheme 1:**
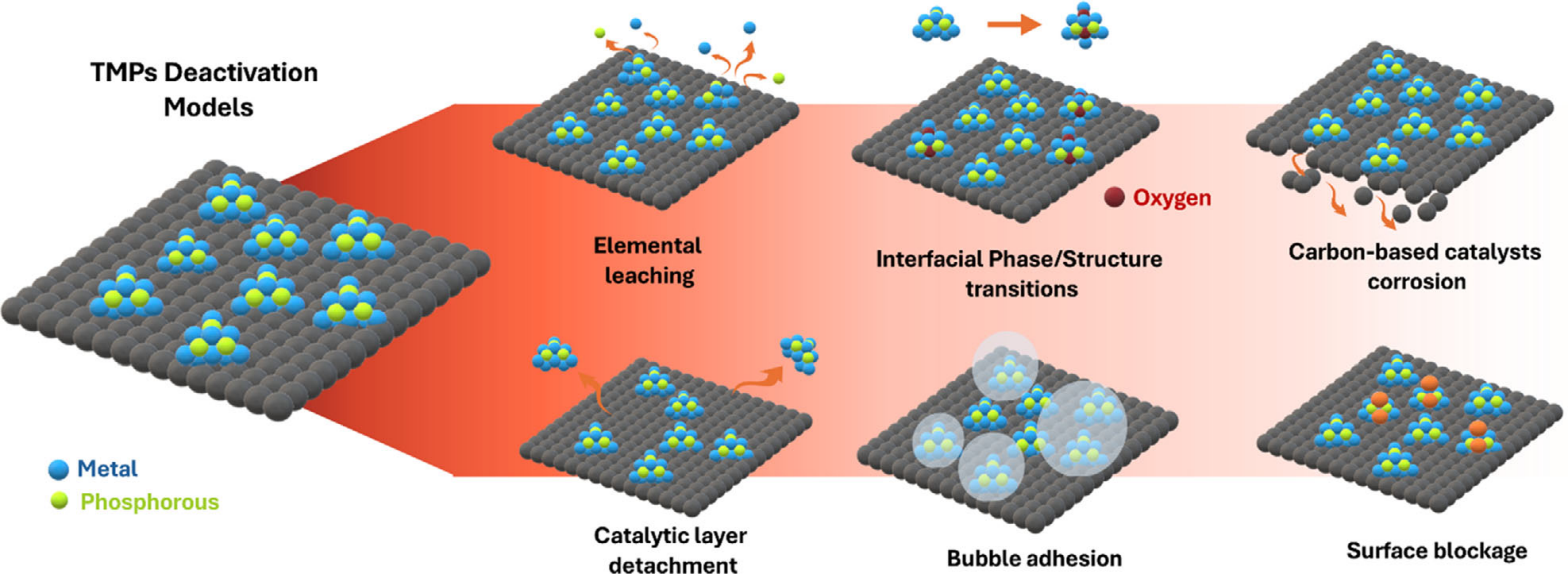
Common degradation mechanisms in TMPs: leaching, detachment, support decay, surface blockage, phase changes, and bubble adhesion.

An electrocatalyst would ideally maintain its consistent performance under operational conditions, preserving the properties without any current loss or potential increase. Although this perfect scenario is rarely achieved with nonnoble catalysts, pursuing long‐term stability is essential for industrial applications.^[^ ^98^ ^]^ Besides high activity and selectivity, pursuing long‐term stability is essential for industrial applications. Hydrogen evolution reactions often occur in complex environments, such as acidic or highly alkaline media (pH ≥ 9) and saline conditions (salinity ≥ 3.5%, seawater). These conditions can lead to the electrocatalyst's chemical transformations (elemental leaching, phase changes, carbon corrosion) and/or physical damage (catalyst detachment, agglomeration, surface blockage, and bubble adhesion) (**Scheme** **1**), compromising long‐term durability and catalytic activity which impacts in the overall performance. This section highlights the most reported degradation mechanisms affecting the stability of TMP‐based electrocatalysts across different pH conditions in HER.

### 3.1 Elemental Leaching

Elemental leaching is a primary cause of catalyst deactivation during water‐splitting electrolysis, and it is a common degradation mechanism in various catalytic processes (photocatalysis, electrocatalysis, and photoelectrocatalysis). In transition metal phosphides, the dissolution of both phosphorus and metal is interconnected and directly influenced by the electrolyte's extreme pH. The predominant leached element varies between acidic and alkaline environments, with acidic conditions primarily promoting metal leaching, while alkaline conditions favor the leaching of phosphorus atoms.^[^ ^99^, ^100^ ^]^ Additionally, factors such as the transition metal, phase, stoichiometry, crystallinity, and unit cell packing also influence the leaching behavior.^[^ ^92^ ^]^

Laursen and coauthors^[^ ^101^ ^]^ investigated the performance of two Ni phosphide phases (Ni_2_P and Ni_5_P_4_) in both acidic and alkaline conditions. Ni_5_P_4_ remained stable during 16 h of H_2_ evolution, Ni_2_P underwent significant corrosion in acidic electrolyte with 50% atomic Ni content loss. These results demonstrate the sensitivity of the lower‐phosphorus‐content catalysts to acidic environments. In contrast, negligible Ni ion detection occurred in alkaline solutions. Unlike metal dissolution, phosphorus loss was more pronounced in alkaline conditions, often occurring as a key step in TMPs oxidation. Su et al.^[^ ^102^ ^]^ found that CoP underwent irreversible changes, losing P atoms from its nanosheets during electrochemical activation in 1 mol L^−^^1^ KOH. This process led to surface reconstruction and the exposure of Co sites, which, over time, led to continued phosphorus loss, resulting in catalyst oxidation and a decrease in HER activity. Hoffman et al.^[^ ^14^ ^]^ also demonstrated that Co_2_P undergoes stoichiometric dissolution during stability testing, with metal and phosphorus atoms leaching in acidic conditions. Su et al.,^[^ ^103^ ^]^ observed a similar effect for alkaline conditions, phosphorus leaching drives the material's degradation into hydroxides, significantly declining in catalyst performance.

Electrode dissolution does not occur only after the start of the reaction, and it typically begins upon immersion in the electrochemical environment. Beyond dissolution during electrolysis, studies have shown that transition metal‐based electrodes can degrade even at open circuit potential, particularly in acidic conditions.^[^ ^104^ ^]^ While OCP leaching varies depending on the type of transition metal phosphide, Mo‐P, despite exhibiting lower dissolution rates than Co‐P during HER electrolysis, is more thermodynamically vulnerable to OCP‐driven dissolution.^[^ ^93^ ^]^ Developing strategies to reduce TMPs deactivation by further dissolution is still essential for achieving high stability in industrial‐scale electrolysis. Recently, sacrificial agents have been used to construct heterostructures with stable materials and pre‐conditioning with low potential during extended HER electrolysis cycles.

### 3.2 Structural Evolution of Interfacial Phases

The effectiveness of nanoscale TMPs as catalysts depends critically on the balance between catalytic activity and stability. High catalytic activity is key for achieving a good reaction rate, and long‐term stability is just as important to maintain the catalyst's longevity and reusability.^[^ ^105^ ^]^ Nanoscale electrocatalysts typically exhibit lower phase stability than bulk materials, often undergoing accelerated surface oxidation and structural reconstruction during long‐term operation.^[^ ^106^, ^107^ ^]^

**Figure 2:**
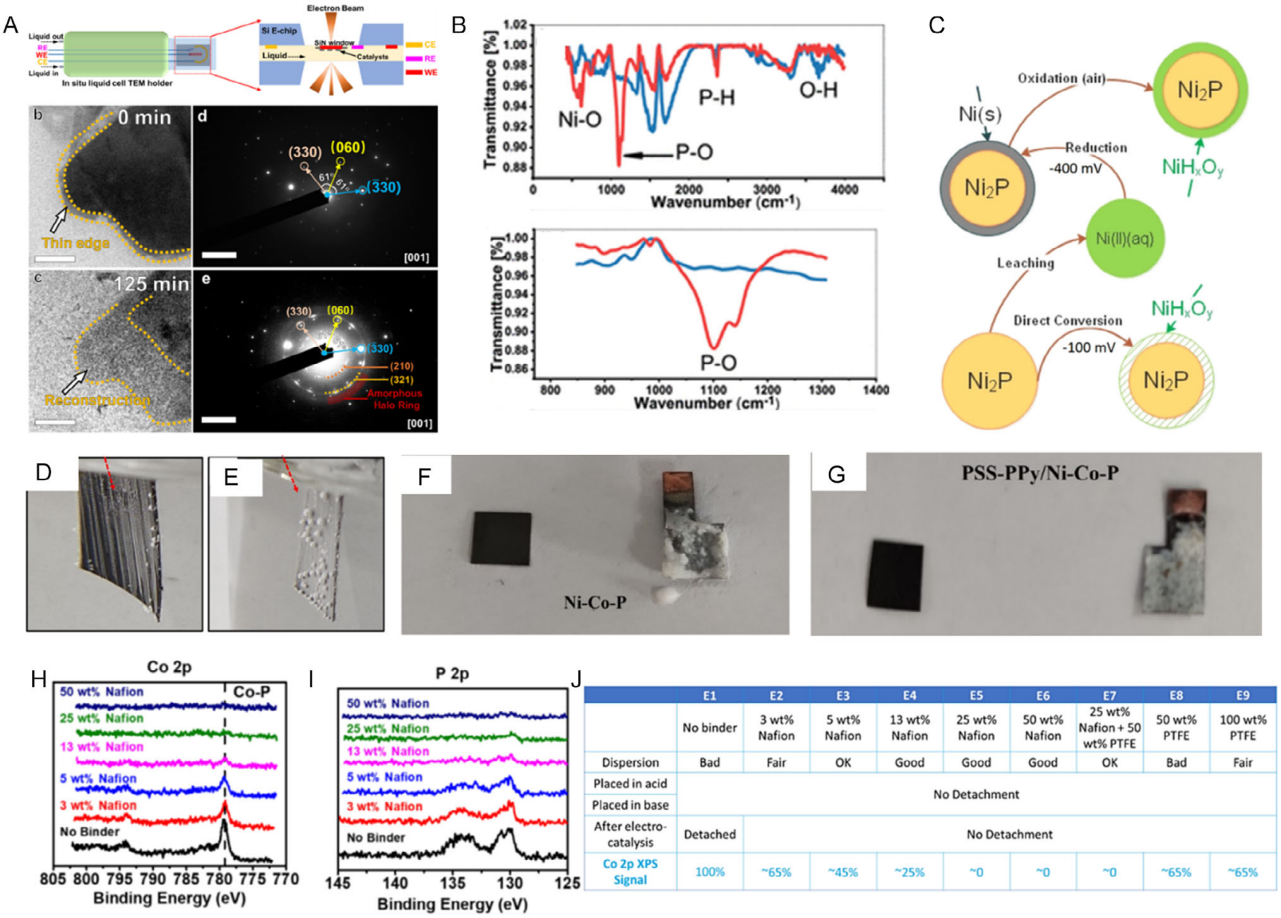
Stability evaluation of Ni_2_P before and after 10 days of electrolysis in 0.50 mol L^−^^1^ H_2_SO_4_ at −400 mV. A) In situ TEM of Ru–NiPS_3_ nanosheets before/after HER shows edge reconstruction; SAED confirmed partial polycrystalline–amorphous transition. Reproduced with permition.^[^ ^109^ ^]^ under CC BY‐SA 2025, Nature Communications. B) FTIR spectra of Ni_2_P before and after the HER test. C) Schematic illustration of Ni_2_P conversion during HER in acidic media. Reproduced with permission.^[^ ^111^ ^]^ Copyrights 2024, American Chemical Society. Optical images of PSS‐PPy‐modified D) and bare Ni*‐*Co‐P E) electrodes during seawater electrolysis, as well as PSS‐PPy‐modified F) and bare Ni*‐*Co‐P G) electrodes after long‐term seawater electrolysis. Reproduced with permission.^[^ ^132^ ^]^ Copyrights 2025, Elsevier. XPS spectra of Co 2p H) and P 2p I) regions of CoP/CNT electrodes prepared with varying weight percentages of Nafion binder. J) Table summarizing the properties of CoP/CNT electrodes with different Nafion and PTFE binder compositions. Reproduced with permission.^[^ ^138^ ^]^ Copyrights 2025, American Chemical Society.

Under ambient, chemical, and electrochemical conditions, phase transitions frequently occur in transition metal catalysts, compromising long‐term stability and contributing to catalyst degradation. While this phenomenon can affect catalysts of various sizes, it is more prevalent in smaller particles due to the increased surface‐to‐volume ratio, which amplifies the influence of surface energy on the catalyst's overall Gibbs free energy.^[^ ^106^, ^108^ ^]^ Using in situ liquid electrochemical TEM, Fu et al.^[^ ^109^ ^]^ observed that Ru–NiPS_3_ nanosheets gradually developed an amorphous layer, primarily along their edges (**Figure** **2**A). Control experiments confirmed that neither the alkaline electrolyte nor the TEM electron beam caused noticeable structural changes. The formation of an amorphous thick layer of ≈7.5 nm contributed to the electrocatalytic process while protecting the nanosheet core from excessive etching and hence deactivation, maintaining the material's morphology and edge integrity, and enhancing long‐term structural stability.

Similar surface restructuring processes have been reported in other transition metal phosphides. Wu and collaborators^[^ ^110^ ^]^ investigated the interfacial phase transition of sulfides and phosphides under ambient conditions during the surface oxidation of the different nanostructures. Due to the high surface energy of the nanostructured Co phosphides (CoP and CoP_2_), they showed fast initial surface oxidation upon exposure to atmospheric air. Even though both oxidized in a few minutes, P‐rich Co‐phosphides exhibited a slower oxidation process than the CoP structure. Other transition metal phosphides based on Mo, Fe, Ni, and Cu displayed substantial initial oxidation of >45% postsynthesis, which limited their storage and further characterization, highlighting the need for preactivation procedures before application. The harsh electrolysis environment, high currents, and aggressive electrolyte environments can also induce phase transitions. Najafpour's group^[^ ^111^ ^]^ performed an extensive in situ study to investigate the phase changes of bulk Ni_2_P material in acidic HER. Significant changes in composition, morphology, and structure were observed in long‐term HER performance, indicating the formation of oxide features resulting from the instantaneous phase modifications of Ni_2_P during electrolysis (Figure 2B). The observed phase modification was driven by Ni leaching from the Ni_2_P structure at a high overpotential (−400 mV). This phenomenon was followed by the redeposition of leached Ni ions as metallic nanoparticles on the electrocatalyst surface (Figure 2C). Subsequent air exposure transformed the metallic deposited layer into an oxidized NiH_*x*_O_*y*_ shell.

While phase transitions did not appear to impact short‐term electrocatalytic activity for HER, they exhibited a significant long‐term challenge, as demonstrated by other studies. For example, Wu et al.^[^ ^112^ ^]^ showed that Fe_0.5_Co_0.5_ P suffers from the rapid dissolution of oxidized P species under high OH^−^ concentrations, leaving behind Fe–OH species. Since iron hydroxide exhibits poor HER activity, restoring Fe_0.5_Co_0.5_ P's activity required an acidic treatment (0.5 mol L^−1^ H_2_SO_4_) to remove the surface oxide layer and expose the bimetallic TMP's surface to the electrolyte. Similarly, Wu et al.^[^ ^112^ ^]^ observed that CoP undergoes a surface transformation into a hydroxide/oxide layer due to P species dissolution in alkaline HER. While Fe_0.5_Co_0.5_ P maintained its current density, CoP exhibited a decline in HER activity compared to its oxidized form. This decline occurs because the leaching of P atoms exposes metallic sites, which can be poisoned by OH^−^ ions in an alkaline medium, reducing available active sites and leading to higher overpotentials during electrolysis.^[^ ^113^ ^]^

### 3.3 Carbon‐Based Catalysts Corrosion

Carbon‐supported TMPs catalysts provide key advantages, including the prevention of nanoparticle agglomeration and particle detachment, as well as improved material conductivity.^[^ ^114^ ^]^ Metal phosphide hybridization with carbon support enhances electron transfer, structural integrity, electrochemical stability, and active surface area.^[^ ^115^ ^]^ This approach has widespread interest in gas‐diffusion electrode applications owing to its simplicity, efficiency, and ability to simulate realistic reaction conditions.^[^ ^116^, ^117^ ^]^ However, the cumulative‐time effect of long‐term operation and frequent startup and shutdown cycles can lead to carbon corrosion,^[^ ^118^, ^119^ ^]^ reducing carbon content and degrading the catalyst layer.^[^ ^120^ ^]^

Although predominantly investigated in fuel cell systems and not yet fully elucidated,^[^ ^121^, ^122^ ^]^ carbon‐based electrodes are susceptible to corrosion under harsh startup and shutdown conditions, such as those encountered in AEMWE stacks.^[^ ^87^ ^]^ Under these circumstances, the formation of a reverse current during system shutdown can induce a self‐discharge process.^[^ ^123^, ^124^ ^]^ Depending on the potential to which the electrodes are exposed, this process can result in irreversible degradation, including catalyst dissolution^[^ ^125^ ^]^ and progressive corrosion of the carbon support.^[^ ^126^ ^]^

While less critical in acidic HER,^[^ ^127^ ^]^ carbon corrosion presents a significant challenge in alkaline environments due to the accelerated electrochemical oxidation of the carbon support, which results in electrical conductivity loss, electrode porosity reduction, isolated pores, catalyst aggregation,^[^ ^128^ ^]^ hindered mass transport, leading to a decline in electrochemical performance. Additionally, it weakens the mechanical integrity of the electrode, ultimately compromising the performance and long‐term stability of supported TMPs catalysts. As the application of supported TMPs expands to more demanding applications, careful consideration of carbon corrosion is imperative, underscoring the need for a thorough evaluation of carbon–support interactions within the electrochemical environment.

### 3.4 Surface Blockage

Chemical deactivation is not the only problem during electrolysis; physical factors like surface blockage, catalyst detachment, and bubble adhesion also play a major role in catalyst deterioration. Surface blockage, in particular, is a significant problem in seawater electrolysis. Despite seawater's abundance and high ionic conductivity,^[^ ^129^ ^]^ the inherent high concentration of alkali species and the rapid pH fluctuations observed near the electrode surface contribute to the precipitation of insoluble hydroxides on the catalyst. This surface deposition results in active site poisoning, reduced charge transfer efficiency, and a subsequent decline in hydrogen evolution performance during prolonged electrolysis.^[^ ^130^, ^131^ ^]^

The use of seawater for H_2_ evolution has been the purpose of many catalyst’ developments.^[^ ^132^, ^133^ ^]^ The great advantage of using seawater is its availability and high ionic conductivity, which prevents the addition of alkaline or acidic supporting electrolytes.^[^ ^129^ ^]^ Just as can be seen, it has an advantageous characteristic, but the high salinity concentration is also a disadvantage that limits the stability of the electrocatalyst for long‐term operation.^[^ ^129^, ^134^ ^]^ For example, Tian and coworkers^[^ ^132^ ^]^ tested the hydrogen evolution performance of PSS‐PPy/Ni*‐*Co‐P and Ni*‐*Co‐P in artificial seawater. Although the authors achieved a similar overpotential to other pH media, the surface of the electrode reacted with the seawater content, and a thick layer of sediments was deposited on the surface of the cathode (Figure 2D,E). The deposited layer acted as a “physical shield” that suppressed catalytic activity, impeding mass transfer and leading to electrode deactivation during the electrolysis (Figure 2F,G).

Catalytic layer blockage is not exclusive to seawater electrolysis but is also prevalent in other water electrolyzers.^[^ ^135^ ^]^ This electrocatalyst blockage can arise from several sources, including the deposition of metal cations, contamination from electrolyte impurities, and the adsorption of organic molecules released during membrane degradation. Even trace levels of impurities can lead to cumulative performance degradation in PEMWE and alkaline water electrolyzers during extended operation. While PEMWE systems mitigate this issue with integrated ion exchange resin purification, the high electrolyte concentration in the AWE system renders this approach impractical. In both cases, the formation of nonactive layers at the electrode‐electrolyte interface results in a reduction in HER performance and a compromise of electrode durability.^[^ ^135^, ^136^ ^]^

### 3.5 Catalytic Layer Detachment

Catalytic layer detachment is a physical deactivation reflected in the catalyst‐current collector interface, which can happen with other deactivation mechanisms, such as elemental leaching and bubble blockage, which induce mechanical stress at the electrode.^[^ ^130^ ^]^ The catalyst detachment is commonly observed in powdery nanostructured electrocatalysts, which, unlike self‐supported TMPs, depend on polymeric binders to adhere to the substrate. However, this approach introduces drawbacks, including uncontrolled microstructures, reduced active surface areas, limited active site access, and undesired interfaces.^[^ ^13^ ^]^

To prevent catalytic layer detachment and ensure the adhesion of nonself‐supported catalysts, the active layer is typically formed on a conductive substrate using an organic binder with low electrochemical background noise. The insolating nature of these binders requires careful control of their content,^[^ ^137^ ^]^ as excessive binder amounts can negatively impact the performance of TMPs catalysts, which can lead to inactivation or even peeling of the catalytic layer. To determine optimal binder proportions, Wu et al.^[^ ^138^ ^]^ Investigated optimal binder proportions for CoP on carbon fiber paper, evaluating catalyst detachment and analyzing results via XPS. As shown in (Figure 2H–J), even with minimal binder amounts, the electrocatalyst often loses the 778.5 eV signal in Co 2p XPS. In contrast, the P 2p signal weakens or vanishes, suggesting that excessive polymeric binder has covered the catalyst surface.

In order to avoid the use of a binder and address the weak interfacial interactions between the catalytic layer and substrate, the adoption of self‐supported catalyst architecture has emerged as a promising strategy for enhancing material adhesion and preventing catalytic layer detachment.^[^ ^139^ ^]^ Self‐supported electrocatalysts are characterized by the direct growth of the catalytic layer on the substrate, which improves electrical conductivity between the catalytic layer and substrate, facilitates better control over surface architecture, and ensures the homogeneous distribution of the active sites.^[^ ^140^ ^]^ The catalyst detachment can be derived from other physical deactivation mechanisms, such as bubble adhesion and surface blockage, that limit the availability of active sites, thereby impacting overall performance.^[^ ^141^ ^]^

### 3.6 Bubble Adhesion

During HER, bubbles nucleated at pores or other electrode defects adhere to the electrode surface. These growing bubbles temporarily obstruct the active sites, limiting mass transport to the electrode surface.^[^ ^142^ ^]^ Strong bubble adhesion results in an increase in ohmic resistance, hindering charge transfer and reducing the overall electrochemical performance.^[^ ^143^ ^]^ The aerophobic properties of an electrode provide superior bubble removal capabilities, facilitating rapid release and enhancing hydrogen evolution kinetics, mitigating degradation and performance loss by bubble adhesion.^[^ ^144^, ^145^ ^]^ During operation, the dynamic of continuous bubble formation, detachment, and imposing mechanical stress on the electrode surface potentially results in material delamination and elemental dissolution, particularly due to the poor adhesion of the catalytic layer at the substrate.

Developing effective surface modification methods to minimize bubble adhesion and promote rapid bubble release is essential for the long‐term operation of electrocatalysts. An interesting approach is to control the hydrophilicity of the electrode. Increased hydrophilicity enhances liquid adhesion while reducing gas bubble adhesion, resulting in a surface with strong aerophobic properties,^[^ ^146^ ^]^ thereby facilitating bubble release through robust interfacial interactions between the electrode and the electrolyte. Another strategy is to regulate surface roughness, as morphology and thickness significantly influence bubble formation and release dynamics.^[^ ^147^, ^148^, ^149^ ^]^

Theoretical simulations of porous electrodes indicate that pore length significantly influences the enhancement effect of bubble detachment on overall transport. Zheng et al.^[^ ^150^ ^]^ found that the pore size and length in aligned porous electrodes jointly determine bubble transport efficiency, which in turn helps maintain a lower overpotential under high current density. Additionally, the average bubble detachment diameter varies with pore diameter and is influenced by electrode thickness. For thinner electrodes (>400 μm), bubble residence time decreases as pore diameter decreases. However, in thicker electrodes (<600 μm), bubbles must travel longer distances before detaching, increasing the detachment diameter with pore size decrease. Overall, in small‐pore electrodes, greater thickness slows bubble transport, preventing efficient bubble expulsion.

## 4 Boosting Durability of Transition Metal Phosphides for Electrocatalysis

Designing efficient electrocatalysts for water electrolysis remains a significant challenge,^[^ ^151^ ^]^ and the development of materials that ensure long‐term performance for HER is an even greater hurdle. The advances in TMPs‐based electrocatalyst design and modification over the years provided valuable experimental and theoretical information concerning the activity and durability under varying pH conditions. Extensive research on reported papers of TMPs incorporating 3d metals (Ni, Co, Fe) and 4d metals (Mo, Ru) has exhibited exceptional stability and electrocatalytic performance, irrespective of the environmental pH. This section provides a critical and comprehensive analysis of the most employed strategies to prevent or mitigate the deactivation of electrocatalysts for HER. The emphasis is placed on how these approaches have been applied specifically to earth‐abundant TMPs, highlighting their effectiveness and unique challenges within this material class.

A practical factor for assessing the practical viability of TMP‐based electrocatalysts is their ability to sustain their electrochemical performance, ensuring their applicability in close‐to‐industry conditions. However, there is a significant limitation in the current literature, which is the emphasis on achieving low overpotential values, but at the expense of systematically assessing the electrode durability of long‐term operation. Consequently, few studies report that TMPs are capable of sustaining long‐term stability for HER. This review provides analysis that also addresses this gap by considering promising TMPs with enhanced durability that exceed the 50‐hour benchmark, even when their performance has been evaluated in only a single pH environment. The studies discussed in this section are summarized in Table S1, Supporting Information.

### 4.1 (Self)‐Supported Catalysts

**Figure 3:**
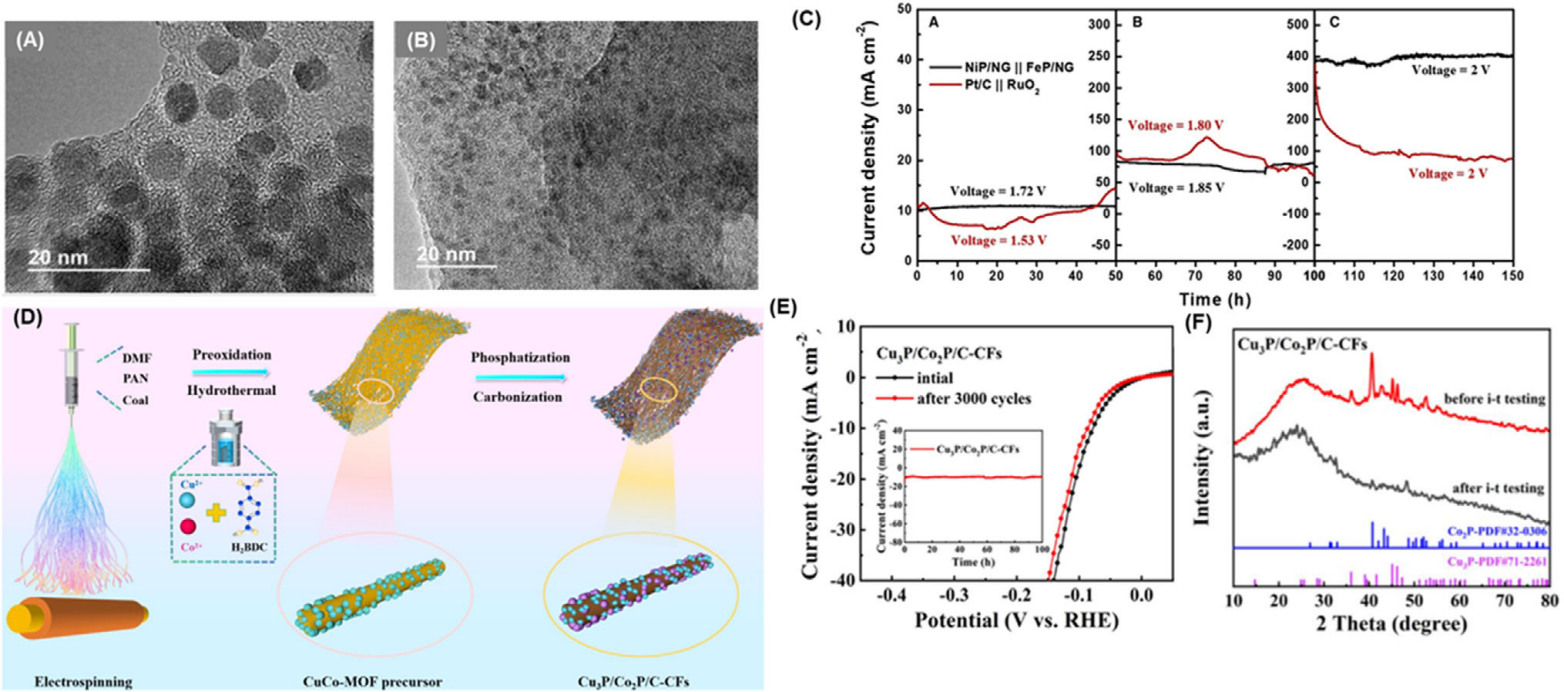
Long‐term water electrolysis performance of NiP/NG and pre‐/post‐electrolysis morphological characterization. HRTEM images of NiP/NG before A) and after B) long‐term water electrolysis show no morphological changes. C) Chronoamperometric analysis of NiP/NG‐FeP/NG (black) and Pt/C‐RuO_2_ (red) during 150 h of continuous operation at increasing voltages (1.7 V, 1.8 V, and 2.0 V) to assess the long‐term stability of the NiP/NG catalyst. Reproduced with permission.^[^ ^153^ ^]^ Copyrights 2025, Elsevier. MOF‐derived Cu_3_P/Co_2_P grown on carbon fibers. D) Schematic illustration of the synthetic route for Cu_3_P/Co_2_P/C‐CFs. E) linear sweep voltammograms before and after long‐term electrolysis. Insert: time‐dependent stability curve of the Cu_3_P/Co_2_P catalyst. F) XRD patterns of Cu_3_P/Co_2_P/C‐CFs before and after long‐term electrolysis. Reproduced with permission.^[^ ^168^ ^]^ Copyrights 2025, Elsevier.

Supported TMPs showed superior improvement in their structural integrity but also optimized their electronic properties, leading to superior catalytic performance. Current high‐performance electrodes often incorporate porous structures to enhance the specific surface area. A class of porous substrates used for fabricating metal phosphide electrodes is carbon‐based support, which has emerged as an effective strategy to mitigate stability loss caused by particle aggregation.^[^ ^152^ ^]^ García's group^[^ ^153^ ^]^ successfully demonstrated this strategy by developing a graphene‐grafted Ni‐P catalyst that maintained its structural integrity and crystallinity after a 150 h stability test at a high current density (>100 mA cm^−^^2^) in an alkaline electrolyte (**Figure** **3**C). The strong interaction between the Ni‐P catalyst and the conductive graphene support enabled higher current densities, even when a polymer binder was used in electrode preparation.

Binder‐loaded electrocatalysts, anchored to conductive substrates by polymeric binders, face weak interfacial bonding, resulting in the catalyst detachment during electrolysis at high‐current levels.^[^ ^154^, ^155^, ^156^, ^157^ ^]^ The effective contact between the electroactive catalyst and the conductive substrate is essential for stable and durable catalytic response, as it enhances electrical conductivity and mechanical stability.^[^ ^13^ ^]^ In this context, self‐supported phosphide catalysts have demonstrated significant promise, offering extended operational lifetimes with minimal stability loss. Based on the findings of this study, this approach is one of the first to produce electrocatalysts capable of sustaining performance beyond 50 h. It has been successfully applied to develop transition metal phosphide electrodes with excellent activity and durability.

3D metallic substrates are widely used to increase surface area and help bubble detachment. Ni and Co foams are common choices as substrates for synthesizing self‐supported phosphides because their surface can be directly converted into phosphides. This conversion is typically achieved through thermal treatment with a phosphorus source in an inert atmosphere and was one of the first strategies developed to produce phosphides with high long‐term stability. For example, Wang and coauthors^[^ ^158^ ^]^ demonstrated the effectiveness of direct thermal phosphidation of Ni foam in producing a multiphase electrocatalyst with stability of up to 72 h at a cathodic current density of 10 mA cm^−^^2^. Although this catalyst does not achieve the lowest overpotential for the hydrogen evolution reaction in acidic media (*η*_10_ = 120 mV), it exhibited exceptional long‐term durability in neutral pH conditions, outperforming other transition metal‐based catalysts reported in the mid‐2010 s.^[^ ^159^ ^]^ Since then, self‐constructed metal phosphide electrodes have been explored using different strategies aiming to reduce electrode resistance and enhance durability for continuous water electrolysis. One of the most used approaches involves investigating different transition metals and their combinations. For instance, Co‐P electrodes show promise for alkaline HER, achieving very low overpotentials (<50 mV for –10 mA cm^−2^).^[^ ^160^, ^161^ ^]^ However, most bare Co‐P electrodes suffer from poor stability during continuous electrolysis in alkaline conditions. In contrast, Ni‐P electrodes have demonstrated good durability for HER in alkaline media (−10 mA cm^−2^ for 96 h),^[^ ^162^ ^]^ but their overpotential is generally higher than that of Co‐based phosphides.

In addition to thermal treatment, electrochemical approaches such as metallic electrodeposition or anodization, followed by thermal treatment, are often employed to refine electrocatalyst morphology further. Although it involves an additional step, the resulting phosphide electrodes usually have better control over the structure and surface properties. Better control over the morphology of transition metal phosphides has proven to be a highly effective strategy for enhancing stability, as demonstrated by Li et al.^[^ ^163^ ^]^ The authors fabricated a self‐assembled porous Co‐P electrode via anodization followed by thermal phosphidation. The electrode exhibited overpotentials of 141, 290, and 328 mV at current densities of –100, –1000, and –1500 mA cm^−^^2^, respectively, and sustained –1 A cm^−^^2^ for 4,000 h with minimal degradation in alkaline electrolyte, highlighting its excellent long‐term durability. Among the TMPs surveyed during the preparation of this review, metal foam supported electrocatalysts^[^ ^164^, ^165^ ^]^ stand out for their exceptionally low overpotentials and remarkable durability, features that position them among the most high‐performing systems reported to date—while acknowledging that cross‐study comparisons must be cautiously approached due to methodological variances.

Morphology‐controlled self‐supported TMPs can also enhance bubble release from the electrode surface, as demonstrated by Yu's group.^[^ ^166^ ^]^ They employed a similar strategy to grow nanoneedles on Ni foam, which were subsequently converted into Ni_2_P, achieving an overpotential of 306 mV at –1,000 mA cm^−^^2^, and good stability (–500 mA cm^−^^2^ for 10 h) in an acidic medium. Although the stability was evaluated for only 10 h, the catalyst exhibited a remarkable “superaerophobic” surface at a high pH value, significantly improving overall electrocatalytic performance by facilitating rapid H_2_ bubble release.

Self‐supported TMPs utilizing carbon‐based materials represent a technique that enables the fabrication of highly porous electrodes while also providing the advantage of producing interconnected structures, which enhance stability and electrochemical performance. One promising approach is electrostatic spinning, which has recently gained attention for producing carbon nanofibers incorporated with metal phosphide catalysts.^[^ ^167^ ^]^ Fan et al.^[^ ^156^ ^]^ demonstrated that this strategy was effective in producing a self‐supported nanosheet array grown on coal‐based nanofibers (Co‐Ni_5_P_4_@C‐CNFs) with enhanced stability during a 100 h stability test in alkaline media, due to their strong mechanical interaction with the carbon‐fiber substrate and the codoping of Co and Ni, which synergistically optimized the electronic structure.

Wang's research group^[^ ^168^ ^]^ utilized a Cu_3_P/Co_2_P/coal‐based carbon fiber electrode for HER in acidic media, which was also fabricated using the electrostatic spinning technique (Figure 3D). The excellent HER performance of the electrode is attributed to the uniform distribution of nanoparticles throughout the carbon fiber support, which increases the availability of electroactive sites in contact with the electrolyte. This design achieved a low overpotential of 83 mV at –10 mA cm^−^^2^ (Figure 3E). Although improved stability was observed after 100 h of I–t testing at –10 mA cm^−^^2^ (insert Figure 3E), the authors reported a tendency for nanoparticle enlargement and aggregation, along with a slight decrease in crystallinity over time (Figure 3F). TMP‐incorporated carbon fibers are being explored as a favorable alternative to enhance the porous structure and stability of catalysts for long‐term operation, they are not effective in mitigating particle aggregation due to crystallinity loss over time^[^ ^168^ ^]^ and interfacial phase transformations.^[^ ^169^ ^]^

### 4.2 Constructing Hybrid Structures and Heterostructures

The stability of TMP‐based catalysts can be improved by incorporating various metals, combining different morphologies and phases. Hybrid and heterostructure metal phosphides create synergistic effects that lower the energy barrier for water dissociation, enhance catalytic activity, and boost stability, especially at high current densities. These hybrid structures provide multiphase interfaces and optimize electronic configurations and usually lead to superior HER performance compared to single‐phase materials.

**Figure 4:**
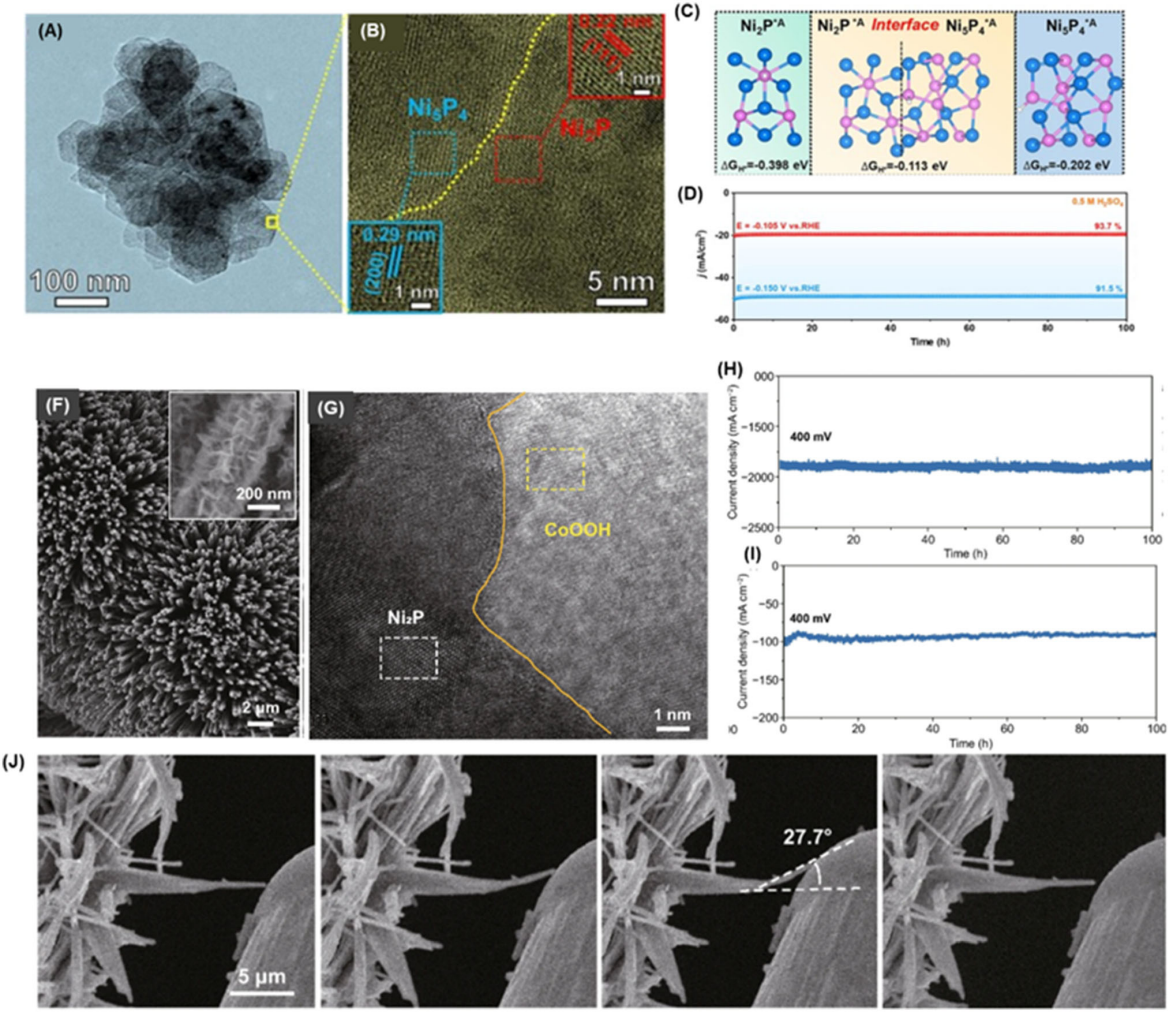
Characterization and Stability of Ni_2_P/Ni_5_P_4_ Heterostructure Nanosheets. A) TEM image of Ni_2_P/Ni_5_P_4_ nanosheets. B) High‐resolution TEM showing the Ni_2_P/Ni_5_P_4_ interface. C) Schematics of Ni_2_P/Ni_5_P_4_ and single phases with H on surface A site. D) I−t curve for Ni_2_P/Ni_5_P_4_ nanosheets in 0.5 mol L^−^^1^ H_2_SO_4_. Reproduced with permission. Copyrights 2025, Elsevier. E) Stability of 2D CoOOH Sheet‐Encapsulated Ni_2_P Tubular Arrays for HER. F) SEM image of the CoOOH‐encapsulated Ni_2_P tubular arrays. G) HRTEM image exhibiting the atomic structure of the material. H) Long‐term stability evaluation of the CoOOH/ Ni_2_P I) arrays at 400 mV for 100 h in alkaline and alkaline + seawater electrolytes. J) In situ SEM measurement of bending deformation and restoration, highlighting the material's mechanical stability during stress. Licensed under CC BY‐ SA 4.0.^[^ ^173^ ^]^ CC 2025, Spring Nature.

Heterostructures based on polymorphic phases of TMPs have demonstrated significant potential. Lyu and coworkers^[^ ^170^ ^]^ developed an interfacial‐engineered Ni_2_P/Ni_5_P_4_ heterostructure that exhibited superior stability compared to single‐phase catalysts (**Figure** **4**A). Theoretical calculations revealed that the Gibbs free energy for hydrogen adsorption (Δ*G*_H_) and water dissociation barriers was significantly reduced due to interfacial synergy (Figure 4B). The Ni_2_P/Ni_5_P_4_ catalyst exhibited excellent stability over 100 h with overpotential retention exceeding 90% across a range of electrolytes (acidic and alkaline media) (Figure 4D). Similarly, Li et al.^[^ ^171^ ^]^ designed a phosphorus‐rich NiCoP and metal‐rich (Fe, Ni)_3_ P heterostructure, achieving a low Schottky barrier due to interfacial electron accumulation. This configuration enabled the catalyst to maintain its performance, with a minimal increase in overpotential (10.7, 30.3, and 15.7 mV) after 50 h in acidic, neutral, and alkaline media, respectively.

**Figure 5:**
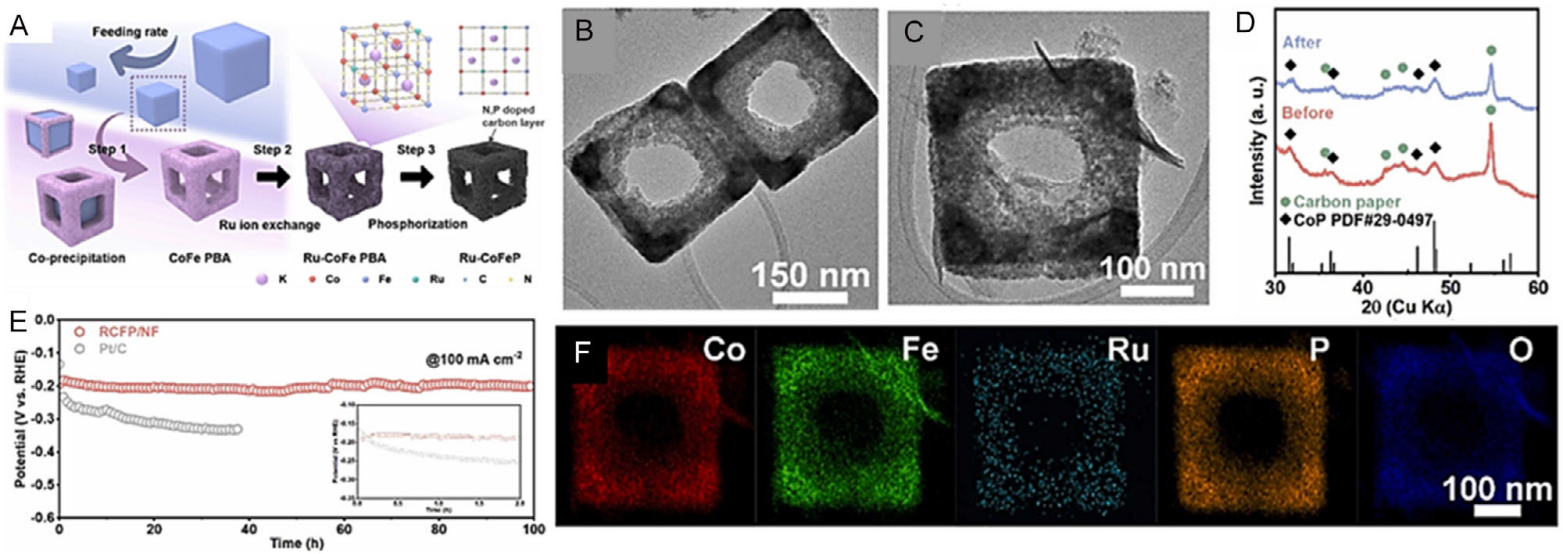
Synthesis, Morphological Characterization, and Stability of Ru‐Doped Co‐Fe‐P Nanoframes. A) Schematic illustration of the synthesis process. B,C) HRTEM images of Ru‐CoFeP nano‐frames before and after constant electrolysis. D) XRD patterns of Ru‐CoFeP pre‐ and post‐electrolysis. E) Constant current electrolysis for stability evaluation. F) STEM‐EDS analysis after electrolysis. Reproduced with permission.^[^ ^190^ ^]^ Copyrights 2025, Elsevier.

The integration of TMPs with metal oxides or hydroxides has shown high effectiveness for high‐current alkaline electrolysis, where the hydroxide phase helps stabilize the phosphide core structure and promotes water dissociation. Chai's group^[^ ^172^ ^]^ developed a CoP/Ni(OH)_2_ heterojunction that achieved *η*_100_ and *η*_500_ values of 108 mV and 175 mV, respectively. The Ni(OH)_2_ layer facilitated the Volmer step and prevented stability loss due to phosphorus leaching in alkaline media, with the electrode maintaining performance for 70 h at 400 mA cm^−^^2^. Zhang et al.^[^ ^173^ ^]^ further optimized this approach by constructing CoOOH‐encapsulated Ni_2_P tubular arrays (Figure 4F,G) through an in situ electrochemical‐driven reconstruction of a bimetallic NiCo core‐shell precursor. The well‐defined 2D sheet‐to‐tube architecture exhibited outstanding HER performance (*η*_10_ = 20 mV). It demonstrated long‐term stability at high current densities (1,200 mA cm^−^^2^ in alkaline electrolyte and 100 mA cm^−^^2^ in seawater + alkaline electrolyte) (**Figure** **5**H). The authors conducted a mechanical stability test on the nanostructured nanotubes, which were interwoven with stacked CoOOH nanosheets, by bending the nanotubes using SEM probes. The SEM images revealed the remarkable flexibility and mechanical stability of the material(Figure 4J). The exceptionally good bending properties were attributed to the nanosheet structure that acts as a buffer layer, allowing the nanotubes to withstand strong impacts caused by hydrogen bubble evolution and electrolyte convection. The unique structure enabled the catalyst to survive mechanical stress, significantly improving its durability and adaptability during long‐term operation. Xin et al.^[^ ^174^ ^]^ developed a heterojunction nanoarray electrode NiFe‐LDH@CoP‐Ni_5_P_4_ that presented an enhanced electronic structure with excellent stability in alkaline electrolytes over 300 h (200 mA cm^−2^), maintaining the surface composition.

Similarly, combining multiple metals and phases introduces new interfaces that tune electronic structures, enhance conductivity, and improve durability. Wu and coworkers^[^ ^175^ ^]^ reported a NiFeP‐MoO_2_ heterostructure, achieving outstanding performance for alkaline electrolysis with stability maintained for 100 h at 300 mA cm^−2^. From density functional theory and density of states calculations, it was revealed that the hybrid structure reduced water dissociation energy and facilitated hydrogen desorption through an increased density of 3 d states. Wen et al.^[^ ^176^ ^]^ also designed a multimetal phosphide with an oxide outer layer. The CeO_2_‐NiCoP_*x*_ electrode was synthesized via thermal phosphidation, resulting in the formation of a CeO_2_ layer that introduced oxygen vacancies and modulated the electronic structure of the metal phosphide. This catalyst achieved remarkable HER performance (*η*_10_ = 39 mV, η_500_ = 205 mV) and sustained operation at 100 mA cm^−^^2^ for 100 h. In situ Raman spectroscopy revealed that the Co and Ce centers acted as key adsorption sites, while oxidized species (e.g., NiO and NiCo_2_O_4_) further contributed to HER activity.

Liu's group^[^ ^177^ ^]^ studied the electronic coupling effect of a Ni_3_P/Ni heterostructure anchored in an N‐doped carbon‐supported catalyst. The authors showed that the Ni_3_P/Ni@N‐CNFs exhibited superior performance compared to the single Ni@N‐CNFs and Ni_3_P@N‐CNFs materials in all the electrolytes (acidic, neutral, and alkaline). The authors demonstrated by electronic orbital hybridization models that introducing the Ni atoms in the Ni_3_P structure induces electron transfer from the metallic Ni toward the P sites, leading to improved electron delocalization and enhancing the H adsorption and water dissociation. Although the overpotentials for HER in the different pH levels were not that low, the Ni_3_P@N‐CNFs/GCE electrode presented good stability at 10 mA cm^−2^, especially in neutral media, remaining stable for 120 h. Mesoporous nitrogen‐doped carbon nanofibers (N‐CNFs) enhance the structural stability and corrosion resistance of Ni_3_P/Ni. While electrospinning has proven to be an effective strategy for developing electrocatalysts, the long‐term stability reported in the literature has predominantly been evaluated at low current densities.

Zhang's group^[^ ^178^ ^]^ developed a NiCoP/NiCoS_X_ heterostructure via electrodeposition, demonstrating the synergistic effect of phosphide‐sulfide hybrids. The optimized interface improved hydrogen adsorption free energy, resulting in improved HER kinetics and overpotentials of 68, 144, and 222 mV at current densities of 10, 100, and 500 mA cm^−^^2^, respectively. The hybrid electrode maintained excellent stability under industrially relevant conditions for 110 h at 500 mA cm^−2^. Carbon‐supported metal phosphides enhance the durability of catalysts for pH‐universal HER and address corrosion issues. A hybrid nanocomposite based on Co‐P phosphide nanoparticles embedded in P‐N‐doped carbon nanotubes prevents Co‐P oxidation and improves durability in both acidic and alkaline media.^[^ ^155^ ^]^ The catalyst maintains stability for 50 h at 20 mA cm^−^^2^ without significant changes in morphology or surface composition, the role of hybrid structures and heterostructures in significantly enhancing HER performance while also improving the durability of TMPs to achieve long‐term stability.

Recently, the hydrogen spillover phenomenon emerged as a promising strategy to promote the catalytic performance of metal‐based catalysts under an alkaline environment.^[^ ^179^, ^180^, ^181^ ^]^ This interaction was investigated by Liu and coworkers^[^ ^182^ ^]^ in a metal phosphide‐metallic particle heterostructure. The resulting Janus‐type structure has distinct electron‐rich and electron‐deficient regions. This interface promoted electron accumulation on the metal nanoparticle, facilitating HER activity. Moreover, during HER in alkaline electrolyte, hydrogen spillover was observed at the catalyst surface, giving rise to a local acidic microenvironment. The catalyst displayed bifunctional activity and excellent long‐term stability, operating at a cell voltage of 1.51 V for 1000 h.

### 4.3 Structure Modulation through Doping

A useful strategy to enhance the long‐term HER performance of metal phosphide electrocatalysts is the modulation of the electronic structure through heteroatom doping. Introducing foreign elements into the host lattice can alter the charge distribution and electronic configuration by modulating the occupation and energy levels of anti‐bonding defect states, which can significantly boost catalytic activity and durability. This approach improves catalytic activity and significantly enhances stability by improving material resistance to degradation, such as metal or phosphorus leaching, across a wide pH range.

While a few single‐phase metal phosphides, such as MoP,^[^ ^183^ ^]^ NiP,^[^ ^184^ ^]^ and FeP,^[^ ^185^ ^]^ have been reported for HER in neutral conditions, their performance is constrained by elevated overpotentials exceeding 100 mV at 10 mA cm^−2^. Indicates inherent inefficiencies in hydrogen adsorption/desorption processes and structural limitations under prolonged operation. To overcome the challenge of high overpotential, researchers have developed heterostructure catalysts incorporating nonmetallic doping and partial phosphidation.^[^ ^132^, ^183^, ^186^, ^187^ ^]^ Yan et al.^[^ ^186^ ^]^ introduced Zn and S into Co‐phosphide (Zn, S–CoP/CPE), demonstrating a substantial reduction in hydrogen adsorption energy (Δ*G*_T_* = 0.07 eV, closer to the thermoneutral point). As a result, the catalyst achieved an outstanding overpotential of 67 mV at 10 mA cm^−^^2^. Moreover, it retained excellent stability for 50 h in neutral electrolytes. However, while Zn and S codoping improved HER kinetics, the long‐term scalability and effects of Zn/S leaching in harsh conditions warrant further investigation.

The major degradation pathway for TMPs in alkaline environments is P leaching and surface reconstruction into oxy/hydroxides, which negatively impacts both stability and performance. Ultralow doping with noble metal has proven highly effective in mitigating these issues. Ru doping stabilizes the TMPs, preventing P leaching and surface reconstruction, and simultaneously enhances the hydrogen evolution reaction performance by optimizing hydrogen binding energies. Chen et al.^[^ ^188^ ^]^ reported a low‐loading Ru‐doped Co‐P nanowire cathode supported in carbon cloth cathode for alkaline H_2_ production, achieving impressively low overpotential (*η*_10_) of 58 mV. DFT calculations revealed that Ru atoms reduce the water dissociation energy, thereby accelerating the HER kinetics Despite impressive activity, structural analysis post‐stability tests (100 h at –50 and –100 mA cm^−^^2^) indicated minor degradation, suggesting that Ru doping alone may not fully stabilize the catalyst under harsh conditions.

Recently, Zhang et al.^[^ ^189^ ^]^ showed that adding Fe into MoRuP significantly enhances its durability in alkaline HER. The Fe‐substituted catalyst Mo_2_Fe_0.8_Ru_0.2_ P retained 96% of its activity after 1000 h at 1.1 A, while undoped Mo_2.8_Ru_0.2_ P suffered an 80% degradation under the same conditions. Although Mo_2.8_Ru_0.2_ P benefits from improved proton dynamics, its stability is compromised due to strong OH^−^ adsorption. The Fe addition mitigates this issue by promoting OH^−^ desorption. A combination of Raman, XRD, XPS, XANES, and DFT analyzes revealed the multimetal synergy in Mo_2_Fe_0.8_Ru_0.2_ P, which modulates the OH^−^ desorption free energy, stabilizes low‐valence metal states, and ultimately enhances the catalyst's long‐term durability for alkaline water splitting.

Further progress was demonstrated by Kim et al.,^[^ ^190^ ^]^ who developed a Ru‐doped Co‐Fe‐P electrocatalyst using a CoFe Prussian blue analog nanoframe (CoFe PBA/NF) as an open‐framework template (Figure 5A). Through Ru ion exchange and subsequent phosphorization, the RuCoFeP/NF retained its nanoframe structure, offering abundant exposed active sites and promoting efficient mass transfer. During phosphorization, the cyanide group (‐CN‐) in PBA was converted into an N, P codoped carbon layer that encapsulated the nanoparticles (Figure 5B). The developed nano‐frames required 216 mV to sustain a current density of 500 mA cm^−^^2^ and demonstrated excellent stability over 100 h at –100 mA cm^−^^2^. However, slight phosphorus (P) dissolution, as identified by ICP‐MS analysis, led to the partial transformation of the material into oxide/hydroxide nanosheets, as observed in HRTEM images (Figure 5C) and XRD pattern (Figure 5D), highlighting the trade‐off between catalytic performance and long‐term phosphorus stability.

While less frequently reported, certain cases suggest that earth‐abundant elements and halogen atoms can enhance TMPs’ stability. For example, Li and coauthors^[^ ^191^ ^]^ synthesized Fe‐doped Co‐P ultrathin nanosheets (Fe‐CoP UNSs/NF) via high‐temperature treatment, achieving excellent stability retention of 90.7% after 50 h electrolysis at −10 mA cm^−2^. TEM analysis confirmed the preservation of the ultrathin nanosheet structure after prolonged operation, highlighting its robustness during long‐term HER testing. Chai and coworkers^[^ ^192^ ^]^ introduced fluorine doping into Co‐Fe‐P, achieving high current densities (e.g., 500 to 3,000 mA cm^−^^2^ at 229.8 to 304.4 mV). Fluorine doping improved electronic conductivity and structural stability, particularly in 6.0 mol L^−1^ KOH, where higher electrolyte conductivity mitigated performance losses.

Additionally, numerous studies are investigating the impact of incorporating earth‐abundant metals with nonabundant elements as multimetal dopants in TMPs to enhance long‐term performance in alkaline electrolytes. Lin et al.^[^ ^193^ ^]^ applied an ion‐exchange method to dope NiCoP nanoneedles with Mo and V species. Mo‐doped NiCoP showed significantly improved stability, attributed to Mo's ability to stabilize the crystal structure under electrochemical conditions. Although the authors evaluated stability performance over a short period, only 20 h, the superior stability of the doped NiCoP was evident. Both Mo‐ and V‐doped NiCoP demonstrated low overpotentials at high current densities (<100 mA cm^−^^2^), highlighting the advantages of strategic dual‐metal incorporation. Guo et al.^[^ ^194^ ^]^ regulated the electronic structure of NiCoP through dual‐metal doping with Mo and W by constructing a W, Mo‐NiCoP nanoarray. The authors achieved a remarkably low overpotential of 294 mV at −1,000 mA cm^−^^2^ with sustained stability over 30 h at a modest potential (≈115 mV). Post‐electrolysis XPS analysis revealed the formation of stable W–P and Mo–P bonds, which provided durable active sites. Liu and coauthors^[^ ^195^ ^]^ extended the dual‐doping strategy to FeP by incorporating Mn and Ni. They observed that Mn doping not only enhanced electrical conductivity and modulated Ni's electronic structure, shifting the d‐band center closer to that of Pt, which enhanced hydrogen adsorption. The resulting Ni‐Mn‐FeP electrode showed excellent stability, maintaining its performance over 120 h at −500 mA cm^−^^2^ while preserving metal‐phosphorus bond integrity at the surface.500 mA cm^−^^2^ while preserving the integrity of metal‐phosphorus bonds at the surface.

Vanadium (V) doping has gained traction due to its multivalence electronic structure. For TMPs doping, Gong's group^[^ ^196^ ^]^ observed that V‐doping into the NiCoP crystal lattice enabled the formation of a lamellar nanosheet array with a large surface area and exposed abundant active sites for HER. Moreover, the V‐NiCoP/CPF electrode demonstrated good structural and electrocatalytic activity in a pH range of 0 to 14 for at least 48 h. Boron is another commonly used doping element, and its incorporation into the TMPs structure was studied by Cao and coworkers.^[^ ^197^ ^]^ They observed that boron doping enhances the catalytic activity of CoP nanoparticles anchored on carbon nanotubes by modulating the Co–P electronic configuration, resulting in thermoneutral hydrogen adsorption with excellent stability sustaining continuous HER for 100 h across all pH electrolytes. The electrode demonstrated robustness by maintaining its electrocatalytic activity after 30 days of exposure to air.

Overall, it is clear that sacrificial doping, as seen with Mn‐modified CoP_*x*,_ provides short‐term protection against degradation. Dual‐metal doping strategies (e.g., Mo, V, W, and Mn) offer a more robust, long‐term solution by stabilizing the crystal structure and optimizing the electronic environment. Moreover, the synergy between carefully chosen dopants enables precise modulation of adsorption energies, conductivity, and d‐band centers, all of which are critical for achieving high catalytic efficiency and prolonged stability under extreme conditions. These advancements collectively emphasize the necessity of combining structural stabilization with electronic optimization to meet the challenges of HER in alkaline media.

### 4.4 Tailoring the Catalyst‐Electrolyte Interface

Self‐restructuring is a common phenomenon in metal phosphide electrodes during HER in alkaline conditions,^[^ ^198^, ^199^, ^200^ ^]^ where interfacial hydroxides form even under reductive potentials. To address this, strategies like engineering metal phosphide/(oxy)hydroxide heterostructures have shown promise in enhancing both catalytic performance and stability. These structures can redistribute surface charge density, lowering the adsorption energy for water molecules while simultaneously protecting the TMP lattice. For instance, Yamauchi and coworkers^[^ ^201^ ^]^ synthesized a Co_2_P–Co_*x*_O_*y*_/CF structure using a one‐step heating method, achieving excellent stability over 100 h of HER operation with negligible potential decay. Similarly, Huang et al.^[^ ^202^ ^]^ demonstrated that low‐temperature phosphidation can selectively form CoP‐Co_*x*_O_*y*_ interfaces, yielding superior HER performance with overpotentials as low as 43 mV at −10 mA cm^−^^2^ and high electrocatalytic and thermal stability. Building on these advancements, Kim et al.^[^ ^164^ ^]^ further advanced the field by synthesizing a crystalline‐amorphous Co‐P hollow nanosheet hybrid, which exhibited remarkable HER activity across a wide pH range. Although the electrode showed good stability in acidic and neutral conditions, it underwent an interfacial transformation in alkaline media, forming a Co‐P/Co(OH)_2_ heterointerface that improved long‐term stability. Despite these advancements, this dynamic restructuring raises questions about long‐term predictability under harsher conditions.

**Figure 6:**
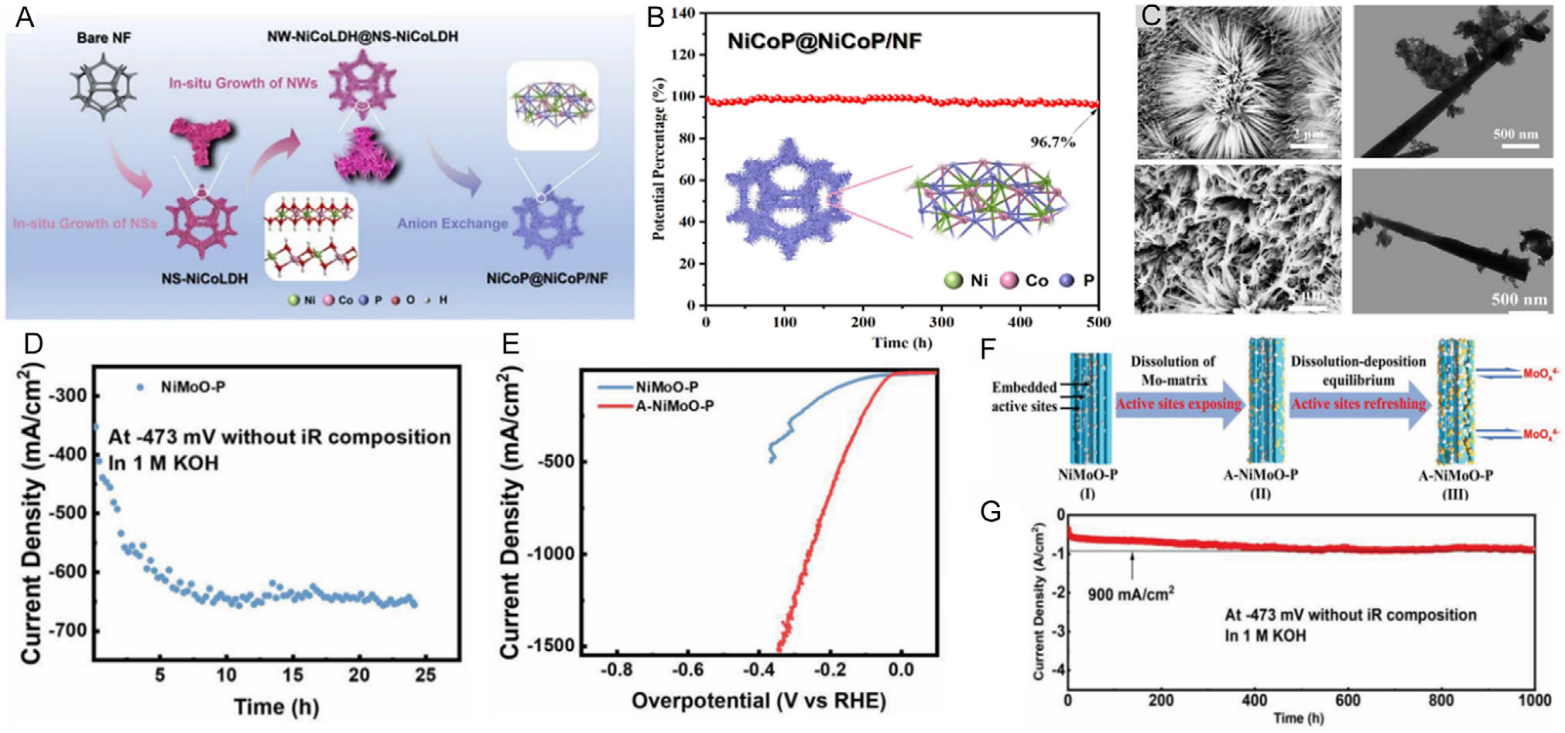
NiCoP nanowires@NiCoP nanosheets on Ni foam: A) Schematic representation of NiCoP@NiCoP/NF electrodes. B) Chronopotentiometry of NiCoP@NiCoP/NF in 1.0 mol L^−1^ at 100 mA cm^−2^ and C) SEM and HRTEM images of the electrode pre‐ and post‐water electrolysis. Licensed under CC BY‐ SA 4.0.^[^ ^127^ ^]^ CC 2025, Catalysts. Schematic illustration and characterization of the in situ refreshing strategy for the NiMoO‐P catalyst during alkaline HER. D) Electrochemical activation of NiMoO‐P catalyst through chronopotentiometry test in 1.0 mol L^−1^ KOH. E) Polarization curves of the catalyst before (NiMoO‐P) and after electrochemical activation (A‐NiMo‐P). F) Schematic illustration of active via a dissolution–redeposition equilibrium. G) Long‐term stability test of the activated electrode. Reproduced with permission.^[^ ^210^ ^]^ Copyrights 2025, Wiley.

Song et al.^[^ ^127^ ^]^ exploited crystal structure heterogeneity to design a NiCoP nanowire@NiCoP nanosheet hybrid on Ni foam (NiCoP@NiCoP/NF). The hybrid structure was synthesized through a two‐step hydrothermal process, where Ni‐Co hydroxides were sequentially grown on a Ni foam substrate at different temperatures to form distinct nanowire and nanosheet morphologies, followed by phosphating at 300 °C. During phosphating, the OH^−^ ions of the precursors are substituted by P^3^^−^ to form the NiCoP catalyst (**Figure** **6**A). The resulting hierarchical structure demonstrated impressive durability, retaining 96.7% of its initial HER overpotential after 500 h at 100 mA cm^−^^2^ (Figure 6B). This remarkable stability was attributed to the composite structure and the presence of amorphous Ni‐/Co‐phosphides, which provided enhanced corrosion resistance. Post‐electrolysis SEM and HRTEM analysis (Figure 6C) revealed that the nanosheets were thicker and larger, with slight aggregation of the nanowires. However, the catalyst was observed to have a significant phase change, which confirmed the structural stability of the catalyst in long‐term operation. The development of crystalline/amorphous heterostructures offers a synergistic advantage: the amorphous regions provide a high density of oxygen vacancies and abundant active sites for enhanced catalytic activity, while the crystalline components ensure long‐term structural integrity.^[^ ^203^, ^204^ ^]^ This combination represents a promising approach for achieving high efficiency and durability in electrocatalysis.

The use of structural templates to enhance catalytic stability and build more robust electrode architectures was also investigated by Hout et al.^[^ ^205^ ^]^ Unlike traditional carbon‐based templates, the authors utilized magnetic NiCoFe alloy nanoparticles to increase the electrode durability by the emerging concept of magnetic field‐assisted electrocatalysis, which represents a novel direction in electrochemistry.^[^ ^206^, ^207^, ^208^ ^]^ External magnetic fields can modulate electrochemical reaction rates, accelerate mass transport, influence the electrode microenvironment, affect magnetoresistance, and guide ion migration within the electrochemical cell. In this study, the magnetic field was employed to improve the long‐term stability of NiCoFeP catalysts. The conductive polymer PEDOT (poly(3,4‐ethylene dioxythiophene) was incorporated not only to enhance the physical stability of the catalyst but also to improve interfacial properties. The synergistic combination of PEDOT coating and magnetic field application increased the electrocatalytic durability of the NiCo‐based HER system. While PEDOT improves the electron structure and surface integrity—resulting in higher activity and extended lifespan, the magnetic field further stabilizes the catalyst by reinforcing its electronic characteristics and forcing bubble removal.^[^ ^207^ ^]^

Kim and coworkers^[^ ^209^ ^]^ proposed an innovative perspective by examining intermittent operation to improve electrode durability in an alkaline electrolyte. Using Mn‐modified CoP_*x*_/Ni plate electrodes, they demonstrated through in situ XANES analysis that sacrificial Mn doping suppresses CoP_*x*_ oxidation by forming protective Mn(OH)_2_ layers. Under on‐off cycling at −200 mA cm^−^^2^, the Mn‐CoP_*x*_ electrode maintained excellent stability, retaining performance for 100 h. Demonstrating the importance of understanding dynamic operational conditions and a promising innovative approach by using sacrificial doping strategies to mitigate oxidation and extend catalyst lifetime.

Building on the theme of dynamic performance, Li et al.^[^ ^210^ ^]^ introduced an in situ refreshing strategy to enhance stability and maintain catalytic activity by creating multilevel active sites. Their design featured an amorphous Mo‐oxide‐embedded Ni_3_(PO_4_)_2_ electrode. During the electrochemical activation process (Figure 6D), Mo dissolution exposed active Ni sites while simultaneously promoting the reduction of Ni phosphate species. This continuous self‐refreshing mechanism (Figure 6) endowed the composite catalyst with remarkable HER performance, achieving ultra‐high current densities of 1,500 mA cm^−^^2^ at an overpotential of 340 mV (Figure 6E) and extraordinary durability, sustaining industrially relevant current densities (≈900 mA cm^−^^2^) for over 1,000 h (Figure 6G).

**Figure 7:**
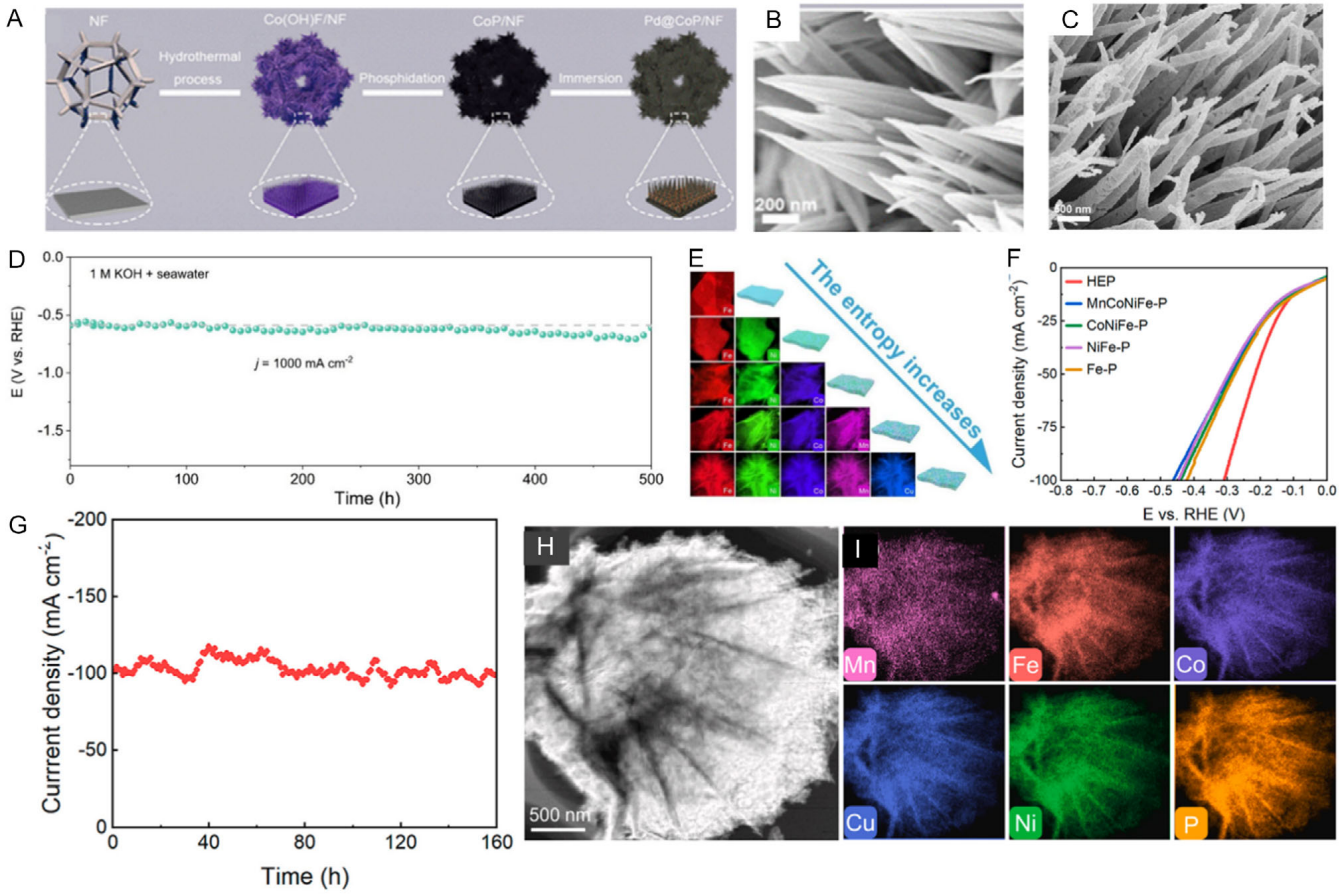
Pd‐decorated CoP nanoneedle arrays on Ni foam: durability and morphological characterization. A) Schematic illustration of the Pd@CoP/NF synthesis process using hydrothermal treatment, followed by phosphatization and Pd immersion. SEM image of the Pd@CoP/NF electrode before B) and after C) electrolysis. D) SEM image of the Pd@CoP/NF electrode after the chronoamperometric test conducted at −1,000 mA cm^−2^ in 1.0 mol L^−1^ KOH + seawater. Reproduced with permission.^[^ ^219^ ^]^ Copyrights 2025, American Chemical Society. FeCoNiCuMnP_*x*_ High Entropy Nanosheets for Enhanced HER Performance in alkaline seawater. E) Elemental mappings of Fe‐, FeNi‐, FeCoNi‐, FeCoNiMn‐, and high‐entropy FeCoNiMnCu precursors. F) Polarization curves registered in 1 mol L^‐1^ KOH with Natural seawater. G) Long‐term chronoamperometric test. TEM image H) and elemental mapping I) of the FeCoNiMnCuP_x_ catalyst after 160 h electrolysis. Reproduced with permission.^[^ ^222^ ^]^ Copyrights 2025, Elsevier.

Durability remains a central challenge for metal phosphide electrocatalysts in alkaline HER due to dynamic surface and interfacial transformations during prolonged operation. All the strategies discussed, including engineering metal phosphide/(oxy)hydroxide heterostructures, combining crystalline and amorphous phases, and self‐regenerating catalytic sites, act directly at the catalyst–electrolyte interface. These approaches promote controlled exposure of active sites while simultaneously enhancing structural stability. Additionally, innovative methods such as sacrificial doping and intermittent operation further modulate interfacial chemistry, mitigating oxidation and extending catalyst lifetime.

Despite these advancements, ensuring long‐term stability under practical conditions remains complex. In this context, machine learning has emerged as an important ally in addressing such challenges by enabling a deeper understanding of interfacial dynamics. Furthermore, recent studies^[^ ^94^, ^95^, ^96^ ^]^ have demonstrated that ML‐trained interatomic potentials, when integrated into ab initio molecular dynamics simulations, offer valuable insight into time‐dependent phenomena like surface reconstruction and electrolyte‐induced corrosion. Together, these advances highlight the potential of data‐driven approaches to accelerate the development of durable and efficient TMP‐based electrocatalysts for hydrogen evolution.

## 5 Unique Aspects of Long‐Term Seawater Electrolysis

Seawater electrolysis offers significant potential for sustainable H_2_ production. However, the complex composition of seawater, including metal cations, anions, solid particles, and microorganisms, poses challenges that limit electrocatalyst activity and durability. For instance, Cl^−^ ions accelerate the corrosion of the electrode substrate and metal electrocatalysts, shortening their operational lifetime.^[^ ^211^ ^]^ Additionally, the presence of calcium and magnesium ions in seawater physically blocks the catalytic site by the formation of insoluble precipitates under alkaline conditions, causing catalyst poisoning. Moreover, metal impurities introduce competing redox reactions during electrolysis and decrease the energy efficiency.^[^ ^134^, ^212^ ^]^ Fortunately, several strategies have been employed to mitigate the precipitate formation, such as pH adjustment using highly concentrated borate or phosphate buffering species to regulate precipitation and minimize the impact of pH fluctuations, and the development of catalysts with a high surface area and abundant active sites, ensuring that even if a small fraction of active sites become deactivated over time, the majority remain available for sustained operation.^[^ ^213^ ^]^

In the HER, pH plays a crucial role in the mechanism and energy efficiency of the process, and natural seawater electrolysis is no exception. Due to the neutral pH, the reaction typically requires higher overpotentials because of the limited availability of H^+^ ions. However, studies have shown that phase engineering in multicomponent electrocatalysts can effectively modulate electronic properties and enhance HER performance,^[^ ^214^ ^]^ even in seawater. For example, Liu et al.^[^ ^215^ ^]^ developed a CoNiP/Co_*x*_P heterostructure on Ni foam that displayed a 290 mV overpotential at −10 mA cm^−^^2^ with remarkable long‐term stability (500 h). The observed stability resulted from the heterointerface, which improved intermediate adsorption and boosted the hydrogen evolution reaction. Likewise, Wu et al.^[^ ^216^ ^]^ explored rare‐earth doping, developing a La_0.17_Mo_0.83_ P electrocatalyst supported on *P*‐doped carbon MXene that improved stability and activity in complex electrolytes. This approach significantly enhanced OH^−^ desorption while maintaining near‐zero H‐adsorption, demonstrating long‐term stability over 400 h.

Research on seawater electrolysis has predominantly concentrated on alkaline seawater due to the more favorable kinetics of both HER and OER under these conditions. Multicomponent electrocatalysts, such as MoO_4_/Ni_2_P/FeP_4_^[^ ^217^ ^]^ and Mn‐doped Ni_2_P/Fe_2_P,^[^ ^218^ ^]^ have demonstrated significant improvements in catalytic activity and stability, with the latter showing up to 200 h of stability in simulated seawater.

Furthermore, morphological engineering of self‐supported TMPs aims to increase specific surface area, a higher density of active sites, improved ion diffusion, and enhanced charge‐transfer kinetics, an effective strategy to optimize phosphide‐based materials for alkaline seawater electrolysis. Wang et al.^[^ ^219^ ^]^ developed Pd‐decorated CoP nanoneedle arrays on Ni foam (Pd@CoP/NF), demonstrating exceptional stability as an HER electrocatalyst in alkaline seawater. The synthesis involved hydrothermal growth of Co(OH)F on Ni foam, followed by phosphidation and immersion in a sodium borohydride and dipotassium hexachloropalladate solution (**Figure** **7**A). Extensive characterization, including SEM (Figure 7B,C), XRD, and XPS, confirmed minimal structural and compositional changes after a 1,000 mA cm^−^^2^ stability test. ICP‐MS analysis showed significantly lower Co leaching from Pd@CoP/NF compared to CoP/NF, attributed to the stabilizing effect of Pd. In an AEM electrolyzer, Pd@CoP/NF outperformed Pt/C/NF, achieving high current densities at lower voltages and maintaining stable operation for over 150 h at 500 mA cm^−^^2^ (Figure 7D).

Another approach to enhancing the long‐term stability of alkaline seawater TMPs is the incorporation of sulfur, resulting in a combined sulfide‐phosphide structure. For example, Wang et al.^[^ ^220^ ^]^ developed a Ni_2_P/NiS_2_ heterostructure that improved HER kinetics and resistance to Cl^−^‐driven corrosion, although long‐term stability was not addressed. Liu et al.^[^ ^221^ ^]^ used interfacial engineering to construct NiFeSP bifunctional catalysts with exceptional long‐term stability (1,000 h at −500 mA cm^−^^2^). The stability resulted from the 3D NiFe framework, which facilitated mass transport and bubble release. The observed metal‐sulfur bond weakening indicated surface changes under reductive conditions. However, challenges remain in regulating the activity and durability of electrocatalysts for long‐term seawater electrolysis. However, challenges remain in regulating the activity and durability of electrocatalysts for long‐term seawater electrolysis. While multicomponent phosphide‐based catalysts show great promise, the lack of in situ studies to understand surface restructuring during cathodic conditions is a critical gap.

High entropy phosphides, composed of five or more elements, have emerged as a potential catalyst class for water‐splitting.^[^ ^222^, ^223^, ^224^ ^]^ The unique interactions between neighboring atoms of different elements create distinctive binding sites, leading to a nearly continuous distribution of relevant adsorption energies and a structure rich in active sites, an essential characteristic for water‐splitting seawater electrodes. Recently, Zhou et al.^[^ ^222^ ^]^ developed a high‐entropy metal phosphide (FeCoNiCuMnP_x_) nanosheet structure using FeCoNiCuMn‐based precursors (Figure 7E), which exhibited excellent HER performance in alkaline seawater due to the high‐entropy effect (Figure 7F). The electrode demonstrated long‐term stability at 100 mA cm^−^^2^ for 160 h. Notably, TEM analysis of the electrocatalyst after extended electrolysis confirmed that it retained its nanosheet morphology (Figure 7I). At the same time, ICP‐MS (Inductively Coupled Plasma Mass Spectrometry) analysis showed no evidence of element dissolution, preserving both the internal crystal structure and elemental distribution.

**Figure 8:**
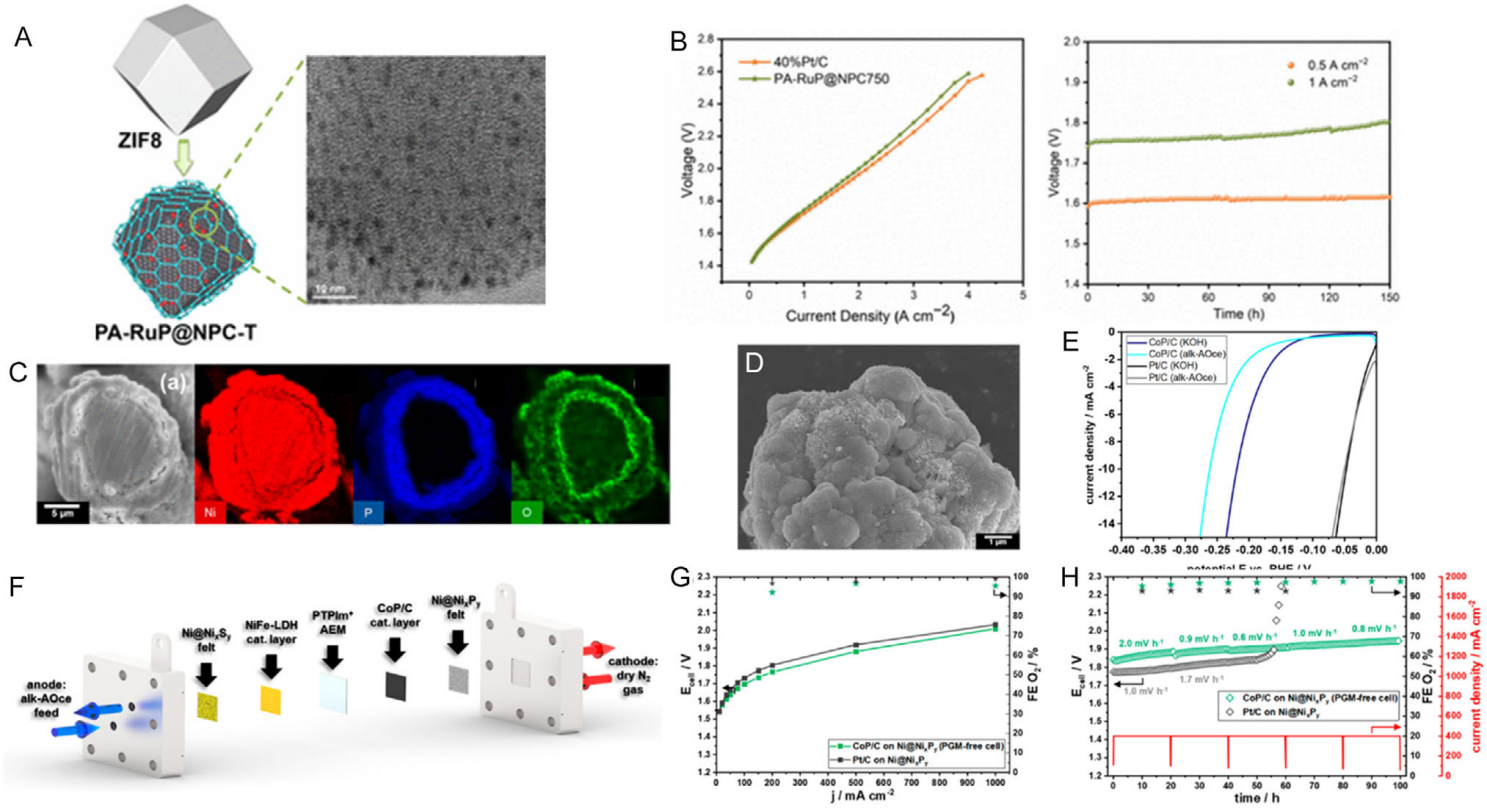
Ruthenium phosphide nanoclusters derived from ZIF8. A) TEM image. B) Comparison of the steady‐state polarization curve and constant curren*t* test of Pt/C and PA‐RuP@NPC‐T in PEM water electrolysis cells at 80 °C. Licensed under CC‐BY‐NC‐ND 4.0,^[^ ^233^ ^]^ 2024. CC, American Chemical Society. C) Cross‐sectional SEM/EDX images show a core‐shell structure with a metallic core and phosphide shell, suggesting phase separation and surface phosphatization. D) SEM image and E,F) Polarization curves CoP/C on 0.1 mol L^−1^ KOH and alkaline ocean water. Single‐cell electrochemical performance at 60 °C in an anodic alk‐AOce feed. G) Polarization curves and H) durability tests of PGM‐free (green) and Pt‐based (gray) MEAs show high Faradaic efficiencies (marked with asterisks). Licensed under CC‐BY‐NC‐ND 4.0,^[^ ^238^ ^]^ 2023 CC, American Chemical Society.

Although seawater electrolysis presents a complex and challenging environment for electrocatalysts over long‐term operation, tailoring electrocatalyst compositions and structures holds significant potential for enhancing performance. The development of protective layers (e.g., metal oxide or metal sulfide coatings), combination of morphologies and phases, and incorporation of multimetal active sites, have shown promising results in improving TMP's stability. Other strategies, such as spin‐state regulation, asymmetric metal site engineering,^[^ ^225^ ^]^ and the synergistic role of P/S, Se ligands, are directly relevant to optimizing the electrocatalytic stability of TMPs and inducing dynamic in situ reconstruction for seawater electrolysis for OER. These findings support the rational design of TMP‐based HER catalysts with improved activity and durability.

## 6 TMPs Stability in Electrolyzer Devices

Evaluating the catalytic activity and durability of metal phosphides in membrane electrode assembly (MEA)‐based systems, such as PEMWEs and alkaline exchange water electrolyzers, is critical for advancing their industrial applicability.^[^ ^226^ ^]^ Conventional stability assessments in single‐ or dual‐compartment electrochemical cells evaluate only the catalyst's intrinsic properties, overlooking interactions within the whole system. Testing industrially relevant electrolyzer devices provides a deeper analysis of fundamental degradation mechanisms. When assessing stability in membrane water electrolyzers, additional factors must be considered beyond those in conventional electrochemical cells, including strong catalyst‐membrane adhesion, sufficient three‐phase boundaries, efficient water and gas transport to the active sites, and excellent conductivity.^[^ ^227^, ^228^ ^]^ In recent years, substantial efforts have been directed toward understanding and improving the performance and stability of transition metal‐based catalysts in electrolyzers, as demonstrated by numerous studies in the literature.^[^ ^229^ ^]^ This section presents the recent advancements in the use of TMP in PEM and AEW electrolyzers. Table S2, Supporting Information, provides a summary of durable TMP‐based electrocatalysts for HER in PEMWE and AEMWE.

### 6.1 TMPs‐Based for PEMWE Devices

Despite the more advanced development of proton water electrolysis technology compared to alkaline water electrolysis, designing low‐cost electrocatalysts that meet the performance requirements for PEMWE (Proton Exchange Water Electrolyzer) operation remains a significant challenge.^[^ ^230^ ^]^ Previous research has established that TMPs‐based catalysts are viable alternatives to platinum‐based materials. A Key limitation of these studies is their reliance on three‐electrode cell systems, which do not accurately reflect the operational conditions of PEMWE. Raising concerns about the TMPs catalyst's application in real‐world settings demonstrates the need for performance evaluation under conditions more representative of actual PEMWE systems.

In early 2019, Jaramillo's group^[^ ^230^ ^]^ successfully scaled up a Co‐P catalyst from lab scale (1 cm^2^) to a commercial‐scale PEM electrolyzer station (86 cm^2^). Although the system exhibited good stability for operation 1,860 mA cm^−2^ for 1700 h at 50 °C and 400 psi., as for the three‐electrode configuration the authors observed Co and P dissolution for the MEA configuration after shutdown and start‐up tests which led to a cell potential. Hence, compared with the Pt/C setup, the Co−P device showed a 12%–18% decrease in power performance. These results represent a significant step forward in enabling the application of metal phosphides in electrolyzer devices.

Wang et al.^[^ ^231^ ^]^ investigated a series of transition metal (Co, Mn, Ni, Cu, Cr, and Mo) incorporation for modulating the hydrogen binding energy of FeP and promoting the HER performance in acidic conditions. The authors verified that the Co‐FeP/C catalyst exhibited the highest stability and catalytic activity, and they also found that the formation of the bimetallic structure significantly reduced elemental leaching during constant electrolysis. PEMWE devices’ performance evaluation showed that the FeCoP/C||IrO_2_ system could reach 800 mA cm^−2^ at 2.0 V with good stability after 1,000 cycles at an accelerated stress test. Jun et al.^[^ ^232^ ^]^ extended this investigation by examining the synergy between Ni and Cu for HER in a PEMWE system. Low‐crystalline NiCuP electrocatalysts with varying Cu/Ni ratios were synthesized on porous transport layers (PTL) using a straightforward electrodeposition method. Their optimized NiCuP–1.2 V/CF/CFP cathode exhibited outstanding single‐cell performance, delivering a current density of 350 mA cm^−^^2^ at 2.0 V and stability testing over 50 h at 1,000 mA cm^−^^2^.

Wang and coauthors^[^ ^233^ ^]^ developed RuP nanoclusters supported on the N, P*‐*co‐doped carbon matrix for HER. The material contains only 8.79 wt% ruthenium. Due to its highly dispersed ultrafine RuP particles (1.55 nm) and uniform distribution on the carbon matrix (**Figure** **8**A), the material exhibits excellent HER activity (*η*_10_ = 13.9 mV) and durability comparable to commercial Pt/C. It was also shown that the RuP phase exhibits superior HER activity and stability to RuP_2_. The improved stability results from a synergistic effect between the N, P‐co‐doped carbon matrix and RuP, whereas the instability originates from the dissolution of the RuP_2_ phase. More importantly, PA‐RuP@NPC750 maintained high activity and stability for over 150 h in PEM water electrolysis systems (Figure 8B). Enhancing the crystallinity of catalysts has been shown to improve the stability of TMPs in the performance of MEA electrolyzers. Duval et al.^[^ ^234^ ^]^ demonstrated that thermally treated Co_2_P@NdC can achieve higher currents, achieving the best performance of 280 mA cm^−^^2^. The incorporation of carbon black further enhanced the total current, with the optimal 1:3 ratio of Co_2_P@NdC450 and stability for 16 h. After stability testing, attempts were made to characterize the tested catalysts. Following stability testing, attempts were made to characterize the tested catalysts. However, due to the minimal catalyst loading on the membrane (10 mg total, equivalent to 2 mg cm^−2^), its strong incorporation into the membrane, and contamination from Nafion, carbon black, and titanium from the gas diffusion layer, detailed characterization proved extremely challenging. The authors claimed that it could not be performed.

### 6.2 TMPs‐Based for PEMWE Devices

Zhao and colleagues^[^ ^235^ ^]^ demonstrated the effectiveness of morphology modulation in enhancing AEM electrolyzer performance. Alkaline water electrolysis remains the primary application for metal phosphides. In their study, the NiFeP_*x*_ catalyst was synthesized by phosphatizing the hydrothermally grown NiFe layered double hydroxide at a low temperature. The NiFeP_*x*_/NF electrode was then employed as the cathode in an anion exchange membrane‐based alkaline electrolyzer operating in 6.0 mol L^−1^ KOH at 60 °C, reflecting commercial catalyst operating conditions. The NiFeOOH||NiFeP_*x*_ system achieved a current density of 10 mA cm^−^^2^ at 1.58 V and demonstrated remarkable stability, sustaining 300 mA cm^−^^2^ for 100 h.

Zhou's group^[^ ^236^ ^]^ developed a vanadium‐doped Ni_2_P/Ni_12_P_5_ heterostructure for alkaline HER. The excellent HER performance of the heterostructure is related to the solid interfacial energy between the Ni‐P phases and the V‐doping that optimized the H_2_ adsorption and water dissociation. AEM electrolyzer potential was performed using the V‐Ni_2_P/Ni_12_P_5_ as the cathode and a NiFeCr LDH as the anode. At 55 °C, the V‐Ni_2_P/Ni_12_P_5_||NiFeCr LDH cell delivers 500 mA cm^−2^ at 1.79 V, which corresponds to 70% energy conversion efficiency much larger than the IrO_2_||(Pt/C) (63% at 1.91 V). Using vanadium and boron as dopants to synergistically accelerate the water dissociation of Ni_2_P, Zhao and coworkers^[^ ^237^ ^]^ developed a highly efficient catalyst for application as the cathode in AEMWE cells for alkaline freshwater and seawater splitting. The NiFeOO > H||B, V‐Ni_2_P electrolyzer required 1.78 and 1.92 V for delivering the current densities of 500 and 1,000 mA cm^−2^ in 1.0 M KOH at 55 °C. The electrolyzer also presented good stability at 500 mA cm^−2^ for 100 h in alkaline freshwater and 30 h for alkaline simulated seawater. From the studies mentioned above, Ni‐P has presented good performances in electrolyzer devices, and electronic modulation by introducing V and Fe enhances the electrode's catalytic activity and durability for applications in water electrolysis devices.

In addition to being used as electrocatalysts for cathodes and anodes in MEA devices, metal phosphides can be used as a porous transport layer for electrolyzers. Frisch et al.^[^ ^238^ ^]^ developed a Ni_*x*_P_*y*_ metallic‐based PTL (Figure 8C). Both Ni‐P PTL and the CoP/C catalyst were prepared via chemical vapor deposition (P‐CVD) (Figure 8D), and the deposition of the Co‐phosphide catalysts was made by spray‐drying on the membrane. The CoP/C and the modified Ni@Ni_*x*_P_*y*_ felt electrodes enhanced HER performance and were further applied in a chalcogenides/phosphide PGM‐free AEMWE device (Figure 8E,H). For the Pt/C‐based MEA, it was observed that there was a significant increase in the cell potential after 55 h of operation in commercially available seawater (Figure 8H). This study demonstrated the feasibility of NiP‐modified Ni felt as a dry cathode assembled for seawater electrolysis.

Demonstrating the feasibility of the doping strategy in an AEMWE electrolyzer, Abisdris et al^[^ ^122^ ^]^ employed theoretical simulations to identify optimal metal dopants for NiP electrodes. Although the DFT calculations were limited to predicting the most stable phase of nickel phosphide in the absence of doping, the approach remains valuable. In doped catalysts, these simulations focus on evaluating the electrochemical activity of the catalysts, not addressing the impact on long‐term stability. Nonetheless, the doped NiP catalysts were experimentally validated and demonstrated remarkable stability. In a 7‐day chronopotentiometry test conducted at a constant current density of 200 mA cm^−2^, the electrolyzer maintained a maximum cell potential of 1.8 V for six days, with only a moderate increase observed during the first 24 h, advancing at a rate of ≈2 mV h^−1^.

## 7 Summary and Outlook

**Figure 9:**
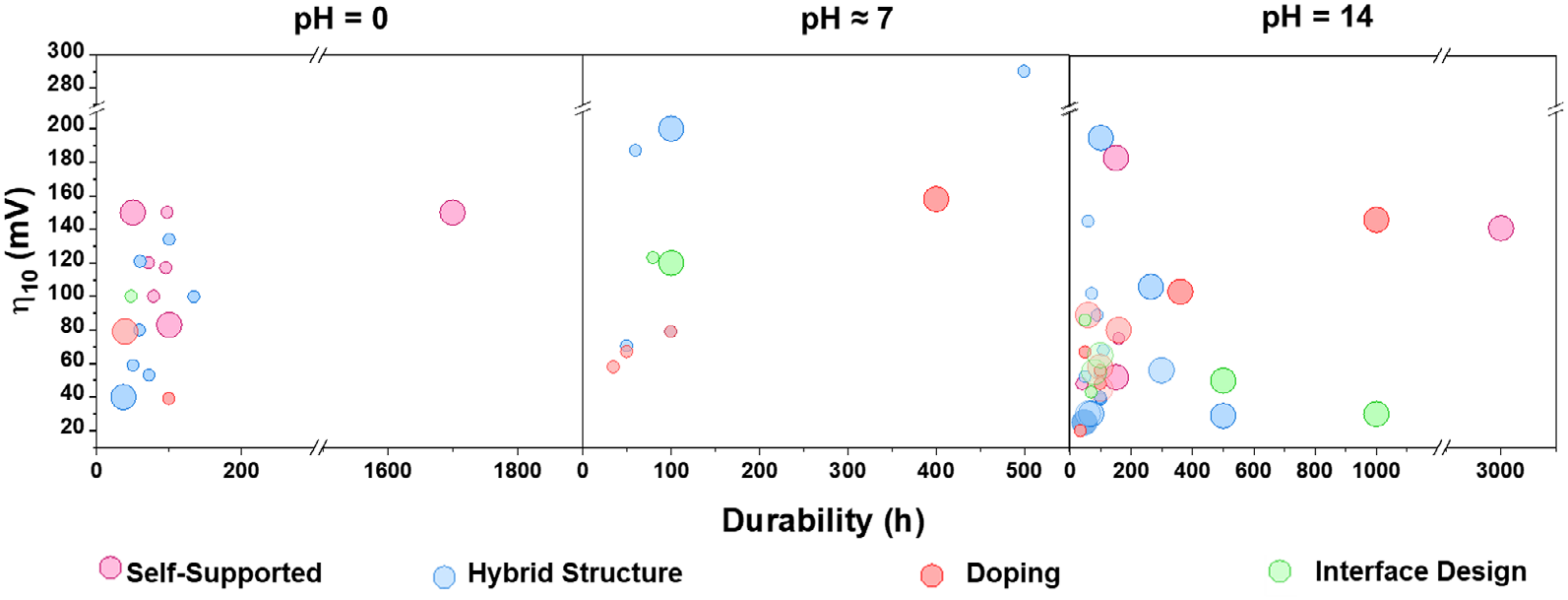
Relationship between the durability (h) and the overpotential (*η*_10_), at different pH conditions (0, 7 and 14). Each point represents the different TMPs electrocatalysts discussed and analyzed in this work, classified by their performance: larger markers indicate high current density (>100 mA cm^−2^). In comparison, smaller markers represent lower current density (<100 mA cm^−2^). The color of each symbol corresponds to the specific strategy employed to enhance catalyst stability.

Over the past decade, transition metal phosphides have emerged as a leading material class for hydrogen production in different pH conditions. Despite this, long‐term stability continues to be a major challenge for industrial applications. This review highlights strategies to not only enhance HER kinetics but also significantly improve catalyst durability in both harsh and neutral environments, such as heteroatom doping, interfacial engineering, morphology control, hierarchical self‐supporting structures, surface wettability tuning, and integration with other abundant transition metal catalysts (e.g., as oxides, sulfides, and MXenes). To better illustrate the HER performance trends, **Figure** **9** presents a comparison of the performance discussed in this review, highlighting the promising strategies for enhanced durability.

From Figure 9, it's possible to have some valuable insights concerning the activity‐stability trend of the TMPs electrocatalysts across the different electrolytic conditions. As we can see, for acidic media, there is a tendency for long‐lasting catalysts to operate at slightly lower overpotentials. Most data points reported in the literature are concentrated at overpotentials for 10 mA cm^−2^ below 100 mV. The data trend observed for neutral environments reflects the challenge associated with the electrocatalysts’ performance at HER in neutral conditions, where interfacial charge transfer is limited. As a result, most catalysts reported in the literature achieve good performance but suffer from rapid deactivation. The most durable catalysts present high overpotential values, demonstrating the necessity of developing electrocatalysts that present lower overpotentials and good durability.

In contrast, as expected, due to the nature of the earth‐abundant transition metals, there is a clear relationship where higher durability is strongly associated with lower overpotential, meaning that the most stable catalysts tend to be more active. These results demonstrate the progress in developing earth‐abundant metal phosphides for alkaline water electrolysis and their suitability for operation in long‐term electrolysis. Several approaches, such as doping, heterostructure design, and protective coatings, have been used to improve the corrosion resistance of TMPs. Still, their effectiveness is ultimately governed by how they modify and stabilize the catalyst–electrolyte interface.

Overall, this trend analysis highlights the importance of jointly evaluating overpotential and durability as key performance descriptors for the HER. Neutral media present significant challenges because of inconsistent performance variability. Still, the clear correlation between durability and activity in alkaline environments offers promising paths for the development of industrially viable electrocatalysts, particularly for applications such as anion exchange membrane water electrolysis (AEMWE). Future studies should focus on deciphering the degradation mechanisms in neutral pH and enhancing structural integrity in highly alkaline environments.

To move beyond laboratory‐scale applications, it is essential to address key issues related to stability, scalability, and integration with real‐world operating conditions. In this context, the following aspects must be considered and further developed to ensure the industrial viability of TMP‐based electrocatalysts: 1) In situ operando characterization. Using in situ operando characterization is essential to comprehend the degradation mechanism and its relationship with the stability of the electrocatalyst during a long‐term reaction. This field still lacks detailed discussion of the actual process that occurs at the interface.^[^ ^239^ ^]^ Studies relying on in situ operando characterization could be used to understand electrode deactivation, leading to a more accurate and rational design that improves the TMP's stability; 2) the substrate significantly influences TPM stability due to the synergetic effect. Beyond serving as the electrode framework, it enhances ion diffusion and electron transfer.^[^ ^240^ ^]^ As a result, catalyst performance can decline significantly if the support substrate undergoes structural degradation. Therefore, robust support development is also an attractive strategy to improve the long‐term performance of transition metal phosphides. Additionally, PEM electrolyzers that are 4.5 times more efficient than other cell configurations^[^ ^241^ ^]^ usually need carbon‐based substrates. This type of substrate is likely to suffer from corrosion and needs suitable treatment to improve its stability; 3) theoretical modeling combined with in situ operando techniques and post‐mortem ex situ can be a powerful approach to understanding metal phosphide modifications, reorganization, and degradation. Theoretical calculations have been extensively used to gain further knowledge of the change in the hydrogen adsorption and water dissociation energies with the electronic modulation strategies. Nevertheless, the simulation studied describes ambient temperature activity and does not reflect the operation in more complex systems like electrolyzers at higher temperatures.^[^ ^242^ ^]^ Machine learning has emerged as a transformative tool for addressing the persistent stability challenges of electrocatalysts for predicting degradation tendencies based on structural and electronic descriptors.^[^ ^243^ ^]^ These models drastically reduce the computational cost of traditional DFT‐based stability assessments while maintaining reasonable accuracy. However, integrating data across multiple scales introduces complexity, as discrepancies between experimental protocols and computational methods necessitate standardized data formats and transformation mechanisms.^[^ ^97^, ^244^ ^]^ By analyzing experimental and computational data, key stability descriptors can be identified, providing a foundation for the rational design of durable TMP catalysts. The integration of ML, artificial intelligence, and automated experimentation offers a promising data‐driven pathway to develop high‐performance and long‐lasting electrocatalysts; 4) low stability in the long‐term performance of metal phosphide materials in “dirty” environments is an issue. Using natural water that contains various impurities such as corrosive ions (Cl^‐^ and SO_4_^−2^) and even microbial organisms leads to blocking active sites and a loss of catalytic activity over time. Coupling the electrolyzer with a purification and desalination pre‐treatment of seawater (reverse osmosis or electrodialysis)^[^ ^245^ ^]^ increases the cost, making it disadvantageous for large‐scale applications. An alternative is developing corrosion‐resistant layers (carbon‐based, polymers, and metal sulfides) on metal phosphides. Some authors have demonstrated that the deposition of a Lewis acid at the transition metal catalyst surface^[^ ^246^ ^]^ is an exciting strategy that facilitates the HER mechanism and prevents catalyst deactivation by precipitate formation during direct seawater electrolysis; 5) experimental setups and parameters standardization for the transition from laboratory to industrial scale. The catalyst stability is intrinsic to the cell configuration, and the well‐established three‐electrode measurements usually do not necessarily translate the same stability behavior into practical electrolyzers. The operating conditions in practical electrolyzers are usually more intense than on a lab scale, and the degradation mechanism can be more severe on the electrocatalyst, affecting the stability performance.^[^ ^247^ ^]^ Then, the transition metal phosphide's stability should be analyzed under higher currents and potential. Additionally, the standardization of electrochemical parameters, such as cell configuration, electrolyte concentration, and electrode geometrical area, would facilitate a comparative analysis between the diverse types of TPM (bimetallic, self‐supported, phosphorus‐rich or metal‐rich, crystalline or amorphous) to refine the intrinsic characteristics that increase the long‐term stability; 6) techno‐economic analysis encompasses the catalyst cost, synthesis, preparation, and stability. In water electrolysis, the H_2_ production cost is greatly influenced by the electrolyzer capital and electricity costs during operations.^[^ ^241^ ^]^ Still, the cost of producing the electrocatalyst should also be addressed since it can be significantly affected by the support type, metal source, activity, and stability. A complete techno‐economic analysis could provide critical insights into the influence of the electrocatalyst on the total electrolysis hydrogen production and the process cost and direct the research toward more efficient preparation methods. Nowadays, there is a crucial need to develop efficient strategies to integrate renewable energy sources into hydrogen production, which requires evaluating catalyst performance under dynamic power supply conditions. Variations in power waveforms, such as square, step, and triangle, significantly influence both catalytic activity and stability.^[^ ^208^, ^248^, ^249^ ^]^ These fluctuations can initially affect the catalyst performance either by improving, such as increasing the surface area, or worsening by promoting the surface oxidation. In this sense, both catalyst design and the characteristics of the power supply profile must be considered. Future research should systematically investigate the effects of parameters such as frequency, amplitude, and voltage variation rate on catalyst durability under fluctuating energy scenarios; and 7) when considering further advanced applications of TMPs, the method of synthesizing and preparing an easily scalable electrode is essential and the lab‐designed electrode's scalability and cost must be considered essential criteria for large‐scale production.^[^ ^250^ ^]^ Developing low‐cost and efficient synthetic methods for metal phosphide electrocatalysts is still challenging, and intensive research is currently ongoing, focusing on the catalyst and the possibility of a scalable preparation process.^[^ ^251^ ^]^ Regarding the scalable electrode preparation process, using phosphide powders does not seem viable since it requires binders, and the self‐assembly methodology is more promising.

These points highlight the main challenges and necessary developments for making TMP‐based catalysts viable for large‐scale, stable, and cost‐effective hydrogen production. Advancements in HER electrocatalysis highlight the potential of self‐restructuring, interface engineering, and dynamic operational strategies to enhance performance and durability. However, further exploration of these mechanisms under dynamic and extreme conditions is crucial to fully realizing their industrial potential.

## Conflict of Interest

The authors declare no conflict of interest.

## Supporting information

Supplementary Material
