# Supplementary material for: Overcoming Stability Hurdles of Transition Metal Phosphides for H2 Evolution: A Review
Source: ChemSusChem. 2025 Sep 11;18(20):e202501388. doi: 10.1002/cssc.202501388 (PMC12548955; doi:10.1002/cssc.202501388)
Supplement: Supplementary file 1 — Supplementary Material [file CSSC-18-e202501388-s001.pdf]

Supplementary Material

Overcoming Stability Hurdles: Strategies for Long-Lasting Transition Metal Phosphides for the Hydrogen Evolution Reaction

Anelisse Brunca da Silva[a, b], Eduardo Arizono dos Reis[a,b,1] , Caue Ribeiro[b*] & Lucia Helena Mascaro[a]

[a]Universidade Federal de São Carlos, Departamento de Química, Rod. Washington Luiz, Km 235, 13565-905, São Carlos, SP, Brazil.

[b]National Nanotechnology Laboratory for Agribusiness (LNNA), EMBRAPA Instrumentação, CP, São Carlos, SP 13560-970, Brazil.

[c]São Carlos Institute of Chemistry, University of São Paulo – USP, São Carlos, São Paulo, Brazil.

[1] Present adress:Universidade Federal de São Carlos, Departamento de Química, Rod. Washington Luiz, Km 235, 13565-905, São Carlos, SP, Brazil.

*Corresponding email address: caue.ribeiro@embrapa.br

Table S1. Summary of Long-term stable TMPs-Based HER Electrocatalysts and Their Key Catalytic Descriptors.

Self-supported TMPs							
**Catalyst**	**Synthetic method**	**Overpotentials mA cm^{-2}@mV**	**Stability**			**Year**	**Ref**
			**CP/retention mV@h@%**	**CA/retention mA cm^{-2}@h@%**	**CV (cycles)**		
NiP/NG	Chitosan-gel + Impregnation + pyrolysis	10@183<br>100@300<br>376@376<br>(1.0 M KOH)	1.72V$_{cell}$@50@–<br>1.80V$_{cell}$@50@–<br>2.00V$_{cell}$@50@–<br>(1.0 M KOH)	–	–	2022	[1]
Cu_3P/Co_2P/C-CFs	Electrospinning + Solvothermal + Phosphatization and Carbonization	10@83<br>(0.5 M H_2SO_4)	–	10@100@94.32<br>(0.5 M H_2SO_4)	3000<br>(0.5 M H_2SO_4)	2025	[2]
Co-Ni_5P_4@C-CNFs	Electrospinning + Hidrothermal + Phosphorization	10@75<br>(1.0 M KOH)	–	10@160@–<br>(1.0 M KOH)	–	2024	[3]
Ni_5P_4-NiP_2/NF	Direct Thermal Phosphidation	10@120<br>100@200<br>(0.5 M H_2SO_4)	–	10@72@–<br>(0.5 M H_2SO_4)	1000<br>(0.5 M H_2SO_4)	2015	[4]
Ni-P/CPE	Electrodeposition + Low-Temperature Thermal Phosphidation	10@117<br>100@250<br>(0.5 M H_2SO_4)	–	–10@96@-<br>(0.5 M H_2SO_4)	1000<br>(0.5 M H_2SO_4)	2016	[5]
Co-P/CF	Anodization + Phosphorization	100@141<br>1000@290<br>1500@328<br>(1.0 M KOH)	–	–1000@3000@–<br>(1.0 M KOH)	–	2020	[6]
FeP/Co_3O_4/CF	Electrodeposition + Phosphidation + Oxidation	10@52<br>100@148<br>1000@356<br>(1.0 M KOH)	–	10 to 1000@150@–<br>(1.0 M KOH)	–	2025	[7]
Ru@Co_2P/CNF	Electrospinning + Solvothermal + Thermal Phosphatization and Carbonization	10@48<br>(1.0 M KOH)	–	50@40@94<br>(1.0 M KOH)	2000<br>(1.0 M KOH)	2023	[8]

Hybrid TMPs Structures							
Catalyst	Synthetic method	Overpotentials mA cm^{-2}@mV	Stability			Year	Ref
			CP/retention mV@h@%	CA/retention mA cm^{-2}@h@%	CV (cycles)		
Ni_2P/Ni_5P_4	Solvothermal + Low-Temperature Thermal Phosphidation	10@134 (0.5 M H_2SO_4) 10@79 (1.0 M PBS) 10@56 (1.0 M KOH)	105@100@93.7 150@100@91.5 (0.5 M H_2SO_4) 135@100@95.6 190@100@94.3 (1.0 M PBS) 85@100@94.4 125@100@92.7 (1.0 M KOH)	–	–	2023	[9]
(Fe, Ni)$_3$P/NiCoP/CP	Hydrothermal + Thermal Phosphidation	10@59;100@155.7 (0.5 M H_2SO_4) 10@70.5; 100@276.5 (1.0 M PBS) 10@52.3; 100@168.3 (1.0 M KOH)	–	50@50@89.3* (0.5 M H_2SO_4) 20@50@69.7* (1.0 M PBS) 20@50@84.3 (1.0 M KOH)	–	2021	[10]
Ni_2P-CoOOH/CF	Hydrothermal + Thermal Phosphidation +Electrodepositon	10@20 (1.0 M PBS) 10@194 (Seawater)	750@100@– (1.0 M PBS) 400@100@– (1.0 M KOH) 400@100@– (Seawater)	1200@100@–1.0 M PBS)	–	2020	[11]
CoP/Ni(OH)$_2$	Molten salt + Thermal Phosphidation + Electrodeposition	100@108 500@175 (1.0 M KOH)	–	400@70@– (1.0 M KOH)	–	2022	[12]
NiFe-LDH@CoP-Ni_5P_4	Hydrothermal + Thermal Phosphidation	10@56 (1.0 M KOH)		200@300@– (1.0 M KOH)		2024	[13]
NiFeP-MoO_2	Hydrothermal + Thermal Phosphidation	10@39 100@110 (1.0 M KOH)	–	300@100@– (1.0 M KOH)	–	2021	[14]
CeO_2-NiCoP$_x$/NCF	Hydrothermal + Thermal Phosphidation	300@166 500@205 (1.0 M KOH)	–	100@100@– 200@50@–	–	2022	[15]
Ni_3P/Ni@N-CNFs /GCE	Thermal Phosphidation	10@121 (0.5 M H_2SO_4) 10@187 (1.0 M PBS) 10@145 (1.0 M KOH	–	10@60@– (0.5 M H_2SO_4) 40@120@–	2000 (0.5 M H_2SO_4,	2022	[16]

				(1.0 M PBS)<br>10@60@–<br>(1.0 M KOH)	1.0 M PBS,<br>1.0 M KOH)		
NiCoP/NiCoSx/NF	Two-step Electrodeposition	10@68<br>100@144<br>500@222<br>(1.0 M KOH)	–	500@110@–<br>(1.0 M KOH)	–	2022	[17]
CoPONPCNTs/CTs	MOF Thermal Phosphidation + Carbonization	10@53.1; 100@224.6;<br>200@358.2 (0.5 M H_2SO_4)<br>10@101.9; 100@191.1;<br>200@271.2 (1.0 M KOH)	–	20@72@75 (0.5 M H_2SO_4)<br>20@72@89.3 (1.0 M KOH)	–	2024	[18]
NiCoFeP@F-NiCoAlFe-LDHs@NiCoFe@poly(3,4-ethylene dioxythiophene) (PEDOT)	Hydrothermal + Thermal Phosphidation	10@106<br>100@320<br>(1.0 M KOH)	227to250@264(1.0 M KOH)	–	–	2024	[19]
**Doped TMPs**							
**Catalyst**	**Synthetic method**	**Overpotentials mA cm^{-2}@mV**	**Stability**			**Year**	**Ref**
			**CP/retention mV@h@%**	**CA/retention mA cm^{-2}@h@%**	**CV (cycles)**		
Zn,S–CoP/CP	Hydrothermal + Thermal phosphidation + Thermal Sulfuration	10@67 (1.0 M PBS)	–	10@50@–<br>(1.0 M PBS)	1000<br>(1.0 M PBS)	2019	[20]
RuCoP/ CC	Hydrothermal + Thermal Phosphidation	10@58<br>(1.0 M KOH)	–	50@100@96.2<br>100@100@88.2<br>(1.0 M KOH)	3000<br>(1.0 M KOH)	2022	.[21]
$Mo_2Fe_{0.8}Ru_{0.2}P$/GCE	Thermal Phosphidation	10@48<br>100@130<br>(1.0 M KOH)	130@100@96	1100@100@96<br>(1.0 M KOH)		2025	
RuCoFeP	Co-precipitation + Ion exchange + Thermal phosphidation	10@112; 100@191;<br>500@219(GCE)<br>10@45; 100@100 (CFP)<br>(1.0 M KOH)	–	100@100@– (GCE)<br>100@100@– (CFP)<br>(1.0 M KOH)	–	2024	[22]
Fe-CoP UNSs/NF	Cyanogel–NaBH4 +	10@67<br>100@148	–	10@50@90.6<br>(1.0 M KOH)	–	2019	[23]

	High-Temperature Phosphidation	(1.0 M KOH)					
F-Co_2P/Fe_2P/ IF	Hydrothermal + Thermal Phosphidation	500@229.8<br>1000@260.5<br>2000@292.2<br>3000@304.4<br>(1.0 M KOH)	–	100@10–<br>500@10@–<br>1000@10@–<br>2000@10@–<br>(for 1.0 and 6.0 M KOH)	1000	2020	[24]
Ni-Mn-FeP	Etching-deposition + Phosphorization	10@103<br>100@177<br>(1.0 M KOH)	–	500@120<br>(1.0 M KOH)<br>500@360<br>(1.0 M KOH)	–	2022	[25]
V-CoP/Ni_2P/NF	Hydrothermal + Thermal Phosphidation	10@79;100@148 (0.5 M H_2SO_4)<br>10@58;100@159 (1.0 M PBS)<br>10@20; 100@100 (1.0 M KOH)	79@35@–(0.5 M H_2SO_4)<br>58@35@–(1.0 M PBS)<br>20@35@–(1.0 M KOH)		10000<br>(0.5 M H_2SO_4)<br>(1.0 M PBS)<br>(1.0 M KOH)	2022	[26]
B-Co/CNT/CG	Thermal Phosphidation	10@39 (0.5 M H_2SO_4)<br>10@79 (1.0 M PBS)<br>10@56 (1.0 M KOH)	47@100@–(0.5 M H_2SO_4)<br>86@100@–(1.0 M PBS)<br>61@100@– (1.0 M KOH)		5000<br>(0.5 M H_2SO_4)<br>(1.0 M PBS)<br>(1.0 M KOH)	2019	[27]

Strategic Interface Design of TMPs

Catalyst	Synthetic method	Overpotentials mA cm^{-2}@mV	Stability			Year	Ref
			CP/retention mV@h@%	CA/retention mA cm^{-2}@h@%	CV (cycles)		
$Co_2P–Co_3O_4$/C/NF		10@86<br>(1.0 M KOH)	–	10@50@90.6<br>(1.0 M KOH)	10000	2023	[28]
Co-P/Co_xO_y/CC		10@43<br>(1.0 M KOH)	72.7@70@–<br>(1.0 M KOH)	–	1000	2022	[29]
CoP/CF		10@48;100@115 (0.5 M H_2SO_4)<br>10@120 (1.0 M PBS)<br>10@55; 100@119 (1.0 M KOH)	–	10and100@100@–(0.5 M H_2SO4)<br>10@100@–(1.0 M PBS)<br>10;100;500@80@–(1.0 M KOH)	10000<br>(0.5 M H_2SO_4)<br>(1.0 M PBS)<br>(1.0 M KOH)	2020	[30]
NiCoP@NiCoP/NF		50@120<br>100@144<br>(1.0 M KOH)	–	100@500@96.7<br>(1.0 M KOH)	–	2023	[31]

A-NiMoO-P/NF	500@170	473@1000@–	–	5000	2024	[32]
Mn-CoP$_x$	10@65	–	200@100(on-off)@–	–	2021	[33]

NF: Ni foam; CFP: Carbon paper; CG: glassy carbon electrode; LDH: Layered Double Hydroxide.

Table S2. Summary of Long-Term Stable TMPs for HER in Seawater.

Catalyst	Synthetic method	Overpotential mA cm^{-2}@mV	Stability			Year	Ref
			CP/retention mV@h@%	CA/retention mA cm^{-2}@h@%	CV (cycles)		
**Natural seawater**							
NiCoP/CoP_x/NF	Electrodeposition + Thermal Phosphorization	10@290	–	10@500@93*	–	2021	[34]
$La_{0.17}Mo_{0.83}P$/PC/MXene	Ultrassonic exfolitaion +Phosphorization + Acid etching	10@158	350 and 450@400@–	–	–	2022	[35]
**Alkaline seawater**							
Ni_2P/NiS_2/NF	Electrodeposition + Thermal Phosphidation	100@188	–	100@48@–	–	2022	[36]
$Ni(PO_3)_2$-CoP_4/$CoMoO_4$/NF	Hydrothermal + Phosphorization	100@94	–	100@60@–	–	2022	[37]
$(Fe_{1-x}Co_x)_2P$/Ni_3N/NF	Direct Nitridation + Thermal Phosphidation	100@109	–	100@40@–	–	2023	[38]
$NiMoO_4$@NiFeP/NF	Hydrothermal + Phosphorization	10@195<br>500@370	–	100@100@–	–	2023	[39]
**Artificial Alkaline seawater**							
$Ni(PO_3)_2$-CoP_4/$CoMoO_4$/NF	Hydrothermal + Phosphorization	100@89	–	100@60@–	–	2022	[37]
NiFePS	Hydrothermal sulfurization + Thermal Phosphorization	10@146<br>100@276<br>500@386	–	500@1000@–	–	2022	[40]

Ni_2P-FeP/FF	In-situ corrosion growth + Thermal Phosphidation	10@89<br>100@178	–	200@90@–	–	2023	[41]
Mn-Ni_2P/Fe_2P	Hydrothermal + Phosphorization	100@308<br>500@425<br>1000@470	–	100@200@–<br>500@120@–	–	2023	[42]
Pd@CoP	Hydrothermal + Impregnation+ Phosphorization	500@206<br>800@305<br>1000@325<br>(1.0 M KOH + Seawater)	–	1000@500@–<br>(1.0 M KOH + Seawater)	–	2024	[43]
FeCoNiCuMnP_x	Hydrothermal + Phosphorization	10@80<br>(1.0 M KOH + Seawater)	320@160@98<br>(1.0 M KOH + Seawater)	–	–	2024	[44]

NF: Ni foam; CFP: Carbon paper.

Table S3: Summary of Durable TMP-Based Electrocatalysts for HER in PEMWE and AEMWE Systems.

Cathode	Anode	Test Cell	Catalyst Loading	Conditions	Electrolyte	Cell voltage@Current Density V_{cell}@A cm^{-2}	Durability A cm^{-2}@h	Year	Ref
**PEMWE Devices**									
CoP/C/ CFP	IrO_x/C/CFP	PEMWE	1 mg cm^{-2}	50°C, 400 psi, 510 mL min^{-1}	Deionized water	2.27@1.86	1.86@1763	2019	[45]
CoFeP/C	IrO_2/C	PEMWE	2 mg cm^{-2}	60°C,	Milli Q water	2.0@0.80	-	2022	[46]
NiCuP	IrO_2/CP	PEMWE/MEA	2 mg cm^{-2}	80°C, 40 mL min^{-1}	Deionized water	2.0@0.35	1@50	2023	[47]
Co_2P/C	IrO_2	PEMWE/MEA	2 mg cm^{-2}	80°C	Milli Q water	2.0@0.44	0.44@16	2024	[48]
PA-RuP@NPC750	IrO_2	PEMWE/MEA	–	80°C, 10 mL min^{-1}	Deionized water	1.60@0.5<br>1.74@1.0	0.50@100<br>1.0@100	2024	[49]
**AEMWE Devices**									
$NiFeP_x$/NF	NiFe LDH/ NF	AEM	–	–	6.0 M KOH	2.15@0.30	0.3@100	2023	[50]
V-Doped $Ni_2P/Ni_{12}P_5$/ NF	NiFeCr/NF	AEM	–	55°C	1.0 M KOH	1.79@0.50	0.50@40	2022	[51]
B, V-Ni_2P	IrO_2	AEM/ MEA	2.0 mg cm^{-2}	55°C (seawater feeding at the cathode and 1.0 M KOH feed at the anode)	1.0 M KOH<br>1.0 M KOH + NaCl	1.75@0.50<br>1.92@1.0	0.50@100 (1.0 M KOH)<br>0.50@30 (1.0 M KOH + 0.5 M NaCl)	2023	[52]
CoP/C	NiFe LDH	$PTPIm^+$ AEM/ MEA	–	60° C, 50 mL min^{-1} (Anodic feed of alkaline seawater), dry N_2 cathode feed (200 mL min^{-1})	Alkaline natural seawater	2.0@1.0	1.0@100	2023	[53]

NF: Ni foam; CFP: Carbon paper; CG: glassy carbon electrode; LDH: Layered Double Hydroxide.

References

[1] J. Hu, L. Peng, A. Primo, J. Albero, H. García, “High-current water electrolysis performance of metal phosphides grafted on porous 3D N-doped graphene prepared without using phosphine” *Cell Rep Phys Sci* **2022**, *3*, DOI 10.1016/j.xcrp.2022.100873.

[2] Y. Feng, M. Lou, L. Wang, S. Wen, Y. Wang, R. Wang, “Bimetallic MOF derived Cu3P/Co2P grown on coal-based carbon fibers as self-supporting electrocatalyst for enhanced hydrogen evolution” *Appl Surf Sci* **2025**, *681*, 161529.

[3] X. Fan, T. Huang, H. Ding, X. Liu, X. Liu, G. Xu, L. Zhang, “Prussian blue analogue-derived Co-Ni5P4 porous nanosheet arrays composite coal-based carbon nanofibers as efficient self-supported electrocatalyst for overall water splitting” *Diam Relat Mater* **2024**, *146*, 111189.

[4] X. Wang, Y. V. Kolen’Ko, X. Q. Bao, K. Kovnir, L. Liu, “One-Step Synthesis of Self-Supported Nickel Phosphide Nanosheet Array Cathodes for Efficient Electrocatalytic Hydrogen Generation” *Angewandte Chemie International Edition* **2015**, *54*, 8188–8192.

[5] X. Wang, W. Li, D. Xiong, D. Y. Petrovykh, L. Liu, “Bifunctional Nickel Phosphide Nanocatalysts Supported on Carbon Fiber Paper for Highly Efficient and Stable Overall Water Splitting” *Adv Funct Mater* **2016**, *26*, 4067–4077.

[6] Y. Li, B. Wei, Z. Yu, O. Bondarchuk, A. Araujo, I. Amorim, N. Zhang, J. Xu, I. C. Neves, L. Liu, “Bifunctional Porous Cobalt Phosphide Foam for High-Current-Density Alkaline Water Electrolysis with 4000-h Long Stability” *ACS Sustain Chem Eng* **2020**, *8*, 10193–10200.

[7] A. A. Alothman, J. H. Shah, M. Un Nisa, S. Mohammad, A. G. Abid, M. Usman, M. Adnan, “Integrated iron phosphides and cobalt oxide electrocatalyst for enhanced hydrogen and oxygen evolution reactions: A study on activity and stability factors” *Int J Hydrogen Energy* **2025**, *107*, 460–468.

[8] R. Zhang, Y. Zhu, Y. Cheng, J. Guan, Q. Zou, B. Guo, M. Zhang, “Ru-modified cobalt phosphide nanoparticles on N-doped carbon nanofibers for efficient hydrogen evolution reaction in alkaline media” *J Alloys Compd* **2023**, *968*, 171889.

[9] C. Lyu, C. Cao, J. Cheng, Y. Yang, K. Wu, J. Wu, W. M. Lau, P. Qian, N. Wang, J. Zheng, “Interfacial electronic structure modulation of Ni2P/Ni5P4 heterostructure nanosheets for enhanced pH-universal hydrogen evolution reaction performance” *Chemical Engineering Journal* **2023**, *464*, DOI 10.1016/j.cej.2023.142538.

[10] W. Li, G. Cheng, S. Peng, M. Sun, S. Wang, S. Han, Y. Liu, T. Zhai, L. Yu, “Tuning hydrogen binding energy by interfacial charge transfer enables pH-universal hydrogen evolution catalysis of metal phosphides” *Chemical Engineering Journal* **2022**, *430*, DOI 10.1016/j.cej.2021.132699.

[11] S. Zhang, W. Wang, F. Hu, Y. Mi, S. Wang, Y. Liu, X. Ai, J. Fang, H. Li, T. Zhai, “2D CoOOH Sheet-Encapsulated Ni2P into Tubular Arrays Realizing 1000 mA cm−2-Level-Current-Density Hydrogen Evolution Over 100 h in Neutral Water” *Nanomicro Lett* **2020**, *12*, DOI 10.1007/s40820-020-00476-4.

[12] F. L. Wang, Y. N. Zhou, J. Y. Lv, B. Dong, X. Y. Zhang, W. L. Yu, J. Q. Chi, Z. X. Wu, L. Wang, Y. M. Chai, “Nickel hydroxide armour promoted CoP nanowires for alkaline hydrogen evolution at large current density” *Int J Hydrogen Energy* **2022**, *47*, 1016–1025.

[13] W. Tan, M. Qin, G. Ma, Z. Fan, X. Li, X. Xin, “Efficient Overall Water Splitting Using NiFe-LDH Nanosheets Modified with CoP-Ni_5P_4 Heterojunction Nanoarray Electrodes” *ACS Sustain Chem Eng* **2024**, *12*, 5139–5149.

[14] X. Wu, J. Li, Y. Li, Z. Wen, “NiFeP-MoO2 hybrid nanorods on nickel foam as high-activity and high-stability electrode for overall water splitting” *Chemical Engineering Journal* **2021**, *409*, DOI 10.1016/j.cej.2020.128161.

[15] S. Wen, J. Huang, T. Li, W. Chen, G. Chen, Q. Zhang, X. Zhang, Q. Qian, K. (Ken) Ostrikov, “Multiphase nanosheet-nanowire cerium oxide and nickel-cobalt phosphide for highly-efficient electrocatalytic overall water splitting” *Appl Catal B* **2022**, *316*, DOI 10.1016/j.apcatb.2022.121678.

[16] C. Fu, L. Feng, H. Yin, Y. Li, Y. Xie, Y. Feng, Y. Zhao, L. Cao, J. Huang, Y. Liu, “Heterogeneous Ni3P/Ni nanoparticles with optimized Ni active sites anchored in N-doped mesoporous nanofibers for boosting pH-universal hydrogen evolution” *Nanoscale* **2022**, *14*, 14779–14788.

[17] W. Han, F. Zhang, L. Qiu, Y. Qian, S. Hao, P. Li, Y. He, X. Zhang, “Interface engineering of hierarchical NiCoP/NiCoSx heterostructure arrays for efficient alkaline hydrogen evolution at large current density” *Nanoscale* **2022**, *14*, 15498–15506.

[18] D. Kong, Q. Xu, N. Chu, H. Wang, Y. Von Lim, J. Cheng, S. Huang, T. Xu, X. Li, Y. Wang, Y. Luo, H. Y. Yang, “Rational Construction of 3D Self-Supported MOF-Derived Cobalt Phosphide-Based Hollow Nanowall Arrays for Efficient Overall Water Splitting At large Current Density” *Small* **2024**, *20*, 2310012.

[19] Z. Q. Hou, R. W. Liu, X. N. Feng, X. L. Jia, K. J. Huang, “Durability over 11 days in electrocatalytic hydrogen evolution reaction via designing a 3D

magnetic electrode and regulating the electronic structure” *Fuel* **2024**, *357*, 130054.

[20] L. Yan, B. Zhang, J. Zhu, Z. Liu, H. Zhang, Y. Li, “Callistemon-like Zn and S codoped CoP nanorod clusters as highly efficient electrocatalysts for neutral-pH overall water splitting” *J Mater Chem A Mater* **2019**, *7*, 22453–22462.

[21] Y. Chen, D. Wang, T. Meng, Z. Xing, X. Yang, “Modulating the Electronic Structure by Ruthenium Doping Endows Cobalt Phosphide Nanowires with Enhanced Alkaline Hydrogen Evolution Activity” *ACS Appl Energy Mater* **2022**, *5*, 697–704.

[22] K. Jang, H. Yoon, J. S. Hyoung, D. S. A. Pratama, C. W. Lee, D. W. Kim, “Enhancement of hydrogen evolution activity by tailoring the electronic structure in ruthenium-heteroatom-doped cobalt iron phosphide nanoframes” *Appl Catal B* **2024**, *341*, 123327.

[23] Y. Li, F. Li, Y. Zhao, S. N. Li, J. H. Zeng, H. C. Yao, Y. Chen, “Iron doped cobalt phosphide ultrathin nanosheets on nickel foam for overall water splitting” *J Mater Chem A Mater* **2019**, *7*, 20658–20666.

[24] X. Y. Zhang, Y. R. Zhu, Y. Chen, S. Y. Dou, X. Y. Chen, B. Dong, B. Y. Guo, D. P. Liu, C. G. Liu, Y. M. Chai, “Hydrogen evolution under large-current-density based on fluorine-doped cobalt-iron phosphides” *Chemical Engineering Journal* **2020**, *399*, DOI 10.1016/j.cej.2020.125831.

[25] Y. Liu, Z. Zhang, L. Zhang, Y. Xia, H. Wang, H. Liu, S. Ge, J. Yu, “Manipulating the d-band centers of transition metal phosphides through dual metal doping towards robust overall water splitting” *J Mater Chem A Mater* **2022**, *10*, 22125–22134.

[26] L. Wang, N. Gong, Z. Zhou, W. Peng, Y. Li, F. Zhang, X. Fan, “Electronic modulation of multi-element transition metal phosphide by V-doping for high-efficiency and pH-universal hydrogen evolution reaction” *Int J Hydrogen Energy* **2022**, *47*, 18305–18313.

[27] E. Cao, Z. Chen, H. Wu, P. Yu, Y. Wang, F. Xiao, S. Chen, S. Du, Y. Xie, Y. Wu, Z. Ren, “Boron-Induced Electronic-Structure Reformation of CoP Nanoparticles Drives Enhanced pH-Universal Hydrogen Evolution” *Angewandte Chemie* **2020**, *132*, 4183–4189.

[28] G. Huang, M. Hu, X. Xu, A. A. Alothman, M. S. S. Mushab, S. Ma, P. K. Shen, J. Zhu, Y. Yamauchi, “ Optimizing Heterointerface of Co 2 P–Co x O y Nanoparticles within a Porous Carbon Network for Deciphering Superior Water Splitting ” *Small Struct* **2023**, 2200235.

[29] M. M. Alsabban, M. K. Eswaran, K. Peramaiah, W. Wahyudi, X. Yang, V. Ramalingam, M. N. Hedhili, X. Miao, U. Schwingenschlögl, L. J. Li, V. Tung, K. W. Huang, “Unusual Activity of Rationally Designed Cobalt Phosphide/Oxide Heterostructure Composite for Hydrogen Production in Alkaline Medium” *ACS Nano* **2022**, *16*, 3906–3916.

[30] H. Yoon, H. J. Song, B. Ju, D. W. Kim, “Cobalt phosphide nanoarrays with crystalline-amorphous hybrid phase for hydrogen production in universal-pH” *Nano Res* **2020**, *13*, 2469–2477.

[31] S. Song, A. Song, L. Bai, M. Duanmu, L. Wang, H. Dong, X. Qin, G. Shao, “Hierarchical Design of Homologous NiCoP/NF from Layered Double Hydroxides as a Long-Term Stable Electrocatalyst for Hydrogen Evolution” *Catalysts* **2023**, *13*, 1232.

[32] Q. Li, C. Chen, W. Luo, X. Yu, Z. Chang, F. Kong, L. Zhu, Y. Huang, H. Tian, X. Cui, J. Shi, Q. Li, Z. Chang, C. Chen, W. Luo, X. Yu, F. Kong, L. Zhu, Y. Huang, H. Tian, X. Cui, J. Shi, “In Situ Active Site Refreshing of Electro-Catalytic Materials for Ultra-Durable Hydrogen Evolution at Elevated Current Density” *Adv Energy Mater* **2024**, *14*, 2304099.

[33] I. S. Kim, H. S. Cho, M. J. Kim, H. J. Oh, S. Y. Lee, Y. K. Lee, C. Lee, J. H. Lee, W. C. Cho, S. K. Kim, J. H. Joo, C. H. Kim, “Sacrificial species approach to designing robust transition metal phosphide cathodes for alkaline water electrolysis in discontinuous operation” *J Mater Chem A Mater* **2021**, *9*, 16713–16724.

[34] D. Liu, H. Ai, M. Chen, P. Zhou, B. Li, D. Liu, X. Du, K. H. Lo, K. W. Ng, S. P. Wang, S. Chen, G. Xing, J. Hu, H. Pan, “Multi-Phase Heterostructure of CoNiP/CoxP for Enhanced Hydrogen Evolution Under Alkaline and Seawater Conditions by Promoting H2O Dissociation” *Small* **2021**, *17*, DOI 10.1002/smll.202007557.

[35] X. Wu, J. Qiu, Z. Wang, “Rare-Earth Doping Transitional Metal Phosphide for Efficient Hydrogen Evolution in Natural Seawater” *Small Struct* **2023**, *4*, 2200268.

[36] H. Y. Wang, J. T. Ren, L. Wang, M. L. Sun, H. M. Yang, X. W. Lv, Z. Y. Yuan, “Synergistically enhanced activity and stability of bifunctional nickel phosphide/sulfide heterointerface electrodes for direct alkaline seawater electrolysis” *Journal of Energy Chemistry* **2022**, *75*, 66–73.

[37] M. Yang, S. Zhang, T. Wang, B. Shi, J. Liu, Y. Tang, Z. Xu, M. T. Sarwar, A. Tang, H. Yang, “Multiple Interface Ni(PO3)2-CoP4/CoMoO4Nanorods for Highly Efficient Hydrogen Evolution in Alkaline Water/Seawater Electrolysis” *ACS Sustain Chem Eng* **2022**, *10*, 12423–12432.

[38] W. Ma, D. Li, L. Liao, H. Zhou, F. Zhang, X. Zhou, Y. Mo, F. Yu, “High-Performance Bifunctional Porous Iron-Rich Phosphide/Nickel Nitride Heterostructures for Alkaline Seawater Splitting” *Small* **2023**, DOI 10.1002/smll.202207082.

[39] D. Guo, Z. Zhao, M.-Y. Zong, C. Fan, W. Zheng, D. Wang, “Engineered Superhydrophilic/Superaerophobic Array Electrode Composed of $NiMoO_4$ @NiFeP for High-Performance Overall Water/Seawater Splitting” *ACS Sustain Chem Eng* **2023**, DOI 10.1021/acssuschemeng.3c01554.

[40] Z. Yu, Y. Li, V. Martin-Diaconescu, L. Simonelli, J. Ruiz Esquius, I. Amorim, A. Araujo, L. Meng, J. L. Faria, L. Liu, “Highly Efficient and Stable Saline Water Electrolysis Enabled by Self-Supported Nickel-Iron Phosphosulfide Nanotubes With Heterointerfaces and Under-Coordinated Metal Active Sites” *Adv Funct Mater* **2022**, *32*, DOI 10.1002/adfm.202206138.

[41] J. Li, M. Song, Y. Hu, C. Zhang, W. Liu, X. Huang, J. Zhang, Y. Zhu, J. Zhang, D. Wang, “A self-supported heterogeneous bimetallic phosphide array electrode enables efficient hydrogen evolution from saline water splitting” *Nano Res* **2022**, DOI 10.1007/s12274-022-4608-8.

[42] Y. Luo, P. Wang, G. Zhang, S. Wu, Z. Chen, H. Ranganathan, S. Sun, Z. Shi, “Mn-doped nickel–iron phosphide heterointerface nanoflowers for efficient alkaline freshwater/seawater splitting at high current densities” *Chemical Engineering Journal* **2023**, *454*, DOI 10.1016/j.cej.2022.140061.

[43] X. Wang, Y. He, Q. Zhang, S. Sun, Z. Li, Z. Cai, C. Yang, M. Yue, M. Zhang, H. Wang, A. Farouk, M. S. Hamdy, J. Hu, X. Sun, B. Tang, “Hydrogen Spillover Boosted Hydrogen Evolution Electrocatalysis over Pd@CoP in Alkaline Seawater” *ACS Mater Lett* **2024**, *6*, 3970–3976.

[44] Y. Zhou, C. Feng, M. Chen, Z. Yang, L. Gao, S. Li, J. Wang, X. Li, Y. Kansha, A. Abudula, G. Guan, “Formation of high entropy phosphide cathode and high entropy oxide anode by morphology remodeling for promoting hydrogen production from overall seawater electrolysis” *Int J Hydrogen Energy* **2024**, *87*, 771–781.

[45] L. A. King, M. K. A. Hubert, C. Capuano, J. Manco, N. Danilovic, E. Valle, T. R. Hellstern, K. Ayers, T. F. Jaramillo, “A non-precious metal hydrogen catalyst in a commercial polymer electrolyte membrane electrolyser” *Nature Nanotechnology 2019 14:11* **2019**, *14*, 1071–1074.

[46] Y. Wang, X. Wang, H. Wei, J. Huang, L. Yin, W. Zhu, Z. Zhuang, “Unveiling the Metal Incorporation Effect of Steady-Active FeP Hydrogen Evolution Nanocatalysts for Water Electrolyzer” *Chemistry - A European Journal* **2023**, *29*, DOI 10.1002/CHEM.202202858.

[47] Y. Jun, J. Kim, S. Y. Kim, S. H. Ahn, “Low-crystalline NiCuP with elemental synergy for hydrogen evolution reaction in proton exchange membrane water electrolyzer” *Fuel* **2023**, *350*, 128737.

[48] S. León Duval, A. Shavel, A. Gual, M. Díaz de los Bernardos, R. Miró, “Co $_2$ P Nanoparticles/N-Doped Carbon Composites as Hydrogen Evolution Electrocatalysts for Proton Exchange Membrane Electrolyzers” *ACS Appl Nano Mater* **2024**, *7*, 17133–17139.

[49] C. Wang, Z. Shi, Y. Wang, G. Peng, W. Luo, M. Xiao, W. Xing, G. Sang, C. Liu, “Ruthenium Phosphide Nanoclusters Dispersed on Zeolitic Imidazolate Framework-8 and Encapsulated in N,P-Codoped Carbon as Electrocatalysts for the Hydrogen Evolution Reaction” *ACS Appl Nano Mater* **2024**, *7*, 25335–25344.

[50] J. Zhao, N. Liao, J. Luo, “Transforming NiFe layered double hydroxide into NiFeP x for efficient alkaline water splitting †” **2023**, DOI 10.1039/d3ta01192e.

[51] T. Zhao, S. Wang, Y. Li, C. Jia, Z. Su, D. Hao, B. jie Ni, Q. Zhang, C. Zhao, “Heterostructured V-Doped Ni2P/Ni12P5 Electrocatalysts for Hydrogen Evolution in Anion Exchange Membrane Water Electrolyzers” *Small* **2022**, *18*, DOI 10.1002/SMLL.202204758.

[52] T. Zhao, S. Wang, C. Jia, C. Rong, Z. Su, K. Dastafkan, Q. Zhang, C. Zhao, “Cooperative Boron and Vanadium Doping of Nickel Phosphides for Hydrogen Evolution in Alkaline and Anion Exchange Membrane Water/Seawater Electrolyzers” *Small* **2023**, DOI 10.1002/SMLL.202208076.

[53] M. L. Frisch, T. N. Thanh, A. Arinchtein, L. Hager, J. Schmidt, S. Brückner, J. Kerres, P. Strasser, “Seawater Electrolysis Using All-PGM-Free Catalysts and Cell Components in an Asymmetric Feed” *ACS Energy Lett* **2023**, *8*, 2387–2394.
